# DHCR7 as a Prognostic and Immunological Biomarker in Human Pan‐Cancer: A Comprehensive Evaluation

**DOI:** 10.1002/cnr2.70376

**Published:** 2025-11-06

**Authors:** Xianghua Wu, Weiwei Zheng, Li Wang, Dan Lin, Zhaoxing Wu

**Affiliations:** ^1^ Department of Neurology, the First People's Hospital of Yuhang District Hangzhou China; ^2^ Department of Laboratory Medicine, the First Affiliated Hospital of USTC, Division of Life Science and Medicine University of Science and Technology of China Hefei China; ^3^ Department of Hematology (Key Laboratory of Cancer Prevention and Intervention China National Ministry of Education), the Second Affiliated Hospital, College of Medicine Hangzhou China; ^4^ Cancer Institute, Zhejiang University Hangzhou China

**Keywords:** DHCR7, immunotherapy, Pan‐cancer analysis, prognostic biomarker, tumor microenvironment

## Abstract

**Background:**

The 7‐Dehydrocholesterol reductase (DHCR7), a critical enzyme catalyzing the final step of the cholesterol biosynthesis pathway, has gained attention for its potential role in tumorigenesis. This study systematically investigated the association between DHCR7 expression and oncogenic processes across multiple cancer types.

**Methods:**

Multi‐omics data were obtained from The Cancer Genome Atlas (TCGA) and Gene Expression Omnibus (GEO) repositories. DHCR7 expression patterns were analyzed using Oncomine, TIMER, and GEPIA platforms. Prognostic significance was assessed via Kaplan–Meier plotter and GEPIA. Tumor stage correlations and immune/molecular subtype associations were evaluated using TISIDB. SangerBox facilitated analysis of DHCR7's associations with immune checkpoint (ICP) molecules, tumor mutational burden (TMB), microsatellite instability (MSI), mutant‐allele tumor heterogeneity (MATH), neoantigen load, and immune cell infiltration.

**Results:**

DHCR7 exhibited significant overexpression in most malignancies, correlating with advanced tumor stage (*p* < 0.05), metastatic progression, and reduced overall survival (HR = 1.34, 95% CI: 1.18–1.52). Strong associations emerged between DHCR7 expression and critical immunomodulatory parameters: positive correlations with ICPs (PD‐L1: *r* = 0.62, CTLA4: *r* = 0.58). Significant links to TMB (*p* = 2.1e−5), MSI (*p* = 4.3e−4), and MATH (*p* = 7.8e−3). Distinct immune infiltration patterns, particularly in bladder carcinoma (BLCA), renal clear cell carcinoma (KIRC), and prostate adenocarcinoma (PRAD). Co‐expression network analysis revealed DHCR7's involvement in immune response regulation (GO:0002764, FDR = 0.003), leukocyte differentiation (GO:0002521, FDR = 0.012), and angiogenesis (GO:0001525, FDR = 0.018).

**Conclusions:**

These pan‐cancer analyses identify DHCR7 as a multifaceted biomarker with dual prognostic and immunotherapeutic relevance. Its involvement in tumor immune microenvironment modulation suggests potential as a therapeutic target.

**Abbreviations**
ACC: adrenocortical carcinoma
BLCA: bladder urothelial carcinoma
BRCA: breast invasive carcinoma
CESC: cervical squamous cell carcinoma and endocervical adenocarcinoma
CHOL: cholangiocarcinoma
COAD: colon adenocarcinoma
DHCR7: 7‐dehydrocholesterol reductase
ESCA: esophageal carcinoma
HNSC: head and neck cancer
ICP: immune checkpoints
KICH: kidney chromophobe
KIRC: kidney renal clear cell carcinoma
KIRC: kidney renal clear cell carcinoma
KIRP: kidney renal papillary carcinoma
LGG: brain lower grade glioma
LIHC: liver hepatocellular carcinoma
LUAD: lung adenocarcinoma
LUSC: lung squamous cell carcinoma
LUSC: lung squamous cell carcinoma
MATH: mutant‐allele tumor heterogeneity
MSI: microsatellite instability
PAAD: pancreatic adenocarcinoma
PCPG: pheochromocytoma and paraganglioma
PRAD: prostate adenocarcinoma
READ: rectum adenocarcinoma
SARC: sarcoma
STAD: stomach adenocarcinoma
STAD: stomach adenocarcinoma
TGCT: testicular germ cell tumors
THCA: thyroid carcinoma
TILS: tumor‐infiltrating lymphocytes
TMB: tumor mutational burden
TME: tumor microenvironment
UCEC: uterine corpus endometrial carcinoma
UVM: uveal melanoma

## 1 Background

The 7‐dehydrocholesterol reductase (DHCR7) enzyme, a critical mediator of cholesterol biosynthesis, catalyzes the conversion of 7‐dehydrocholesterol (7‐DHC) to cholesterol, serving as a metabolic hub with pleiotropic implications in development, immunity, and cancer [1]. While DHCR7 mutations are well‐documented in Smith‐Lemli‐Opitz syndrome (SLOS), an autosomal recessive disorder characterized by developmental abnormalities, intellectual disability, and immune dysfunction [2, 3]. Emerging research underscores its multifaceted role in oncogenesis, immune evasion, and therapy resistance. For example, DHCR7 overexpression correlates with poor prognosis in cervical, gastric, breast, and bladder cancers [4, 5, 6, 7], while loss‐of‐function mutations (e.g., rs104886035/rs104886038) disrupt cholesterol metabolism to suppress tumor growth [5]. Paradoxically, DHCR7 deficiency also confers resistance to ferroptosis via 7‐DHC accumulation, revealing a survival advantage in certain cancers [8, 9]. Beyond metabolism, DHCR7 also intersects with immune signaling—cholesterol depletion via DHCR7 inhibition enhances antiviral responses by upregulating interferon regulatory factor 3 (IRF3) activity [10, 11]. Wang et al. demonstrated that DHCR7‐mediated cholesterol synthesis represses IRF3‐driven antiviral pathways, proposing DHCR7 inhibitors as potential immune adjuvants [12]. Despite these advances, existing studies remain fragmented, focusing narrowly on isolated cancer types or mechanistic pathways; no systematic study has explored DHCR7's pan‐cancer immunological relevance or its potential as a biomarker for immunotherapy response, leaving a pivotal knowledge gap in immuno‐oncology. In this study, we conduct a comprehensive multi‐omics analysis of DHCR7 across 33 cancer types, integrating bulk and single‐cell RNA sequencing, immune deconvolution, and clinical outcome data to systematically evaluate its prognostic significance and immunological relevance. We resolve conflicting reports on DHCR7's dual roles in tumor progression (pro‐ vs. anti‐tumor effects) and immune regulation, elucidating its impact on immune checkpoint modulation and tumor microenvironment (TME) remodeling. With a focused investigation of urogenital cancers, we delineate DHCR7's immune‐related mechanisms by assessing its correlations with immune subtypes, tumor‐infiltrating lymphocyte (TIL) dynamics, and response to immune checkpoint inhibitors (ICIs). Finally, we examined the protein expression level of DHCR7 in renal epithelial carcinoma cell lines and further demonstrated its involvement in regulating malignant cell proliferation by establishing DHCR7‐knockdown cell lines. Collectively, our study bridges a critical knowledge gap by presenting the first pan‐cancer, immune‐centric exploration of DHCR7, unveiling its dual function as a metabolic regulator and immunomodulator with potential therapeutic implications.

## 2 Methods

### 2.1 Oncomine Analysis

The Oncomine database (https://www.oncomine.com) is a bioinformatics tool for collecting, standardizing, analyzing and delivering cancer transcriptome data to the biomedical research community. It was used to compare the transcription levels of DHCR7 between cancer specimens and para‐carcinoma tissue. In Oncomine, Student's *t*‐test is generated for two‐class differential expression analyses. In the present study, *p* < 0.01 and an absolute fold‐change ≥ 1.5 were selected as the cut‐off values to analyze the gene expression chart of each DHCR7 family member.

### 2.2 Gene Expression Profiling Interactive Analysis (GEPIA) Database

GEPIA (http://gepia.cancer‐pku.cn/) is a web tool that provides fast and customizable functionalities based on data from The Cancer Genome Atlas (TCGA; http://tcga‐data.nci.nih.gov/tcga/) and the Genotype‐Tissue Expression project (GTEx; http://www.gtexportal.org/home/index.html). Differential analysis was performed using one‐way ANOVA, using disease state or tumor stage as the variable for calculating differential expression. In our study, GEPIA was used to exhibit the differential expression of DHCR7 between pan cancer and the related para carcinoma tissues, and the association between the expression of DHCR7 and tumor stages in patients.

### 2.3 The Human Protein Atlas Database

The Human Protein Atlas (https://www.proteinatlas.org/) is a database of immunohistochemistry (IHC)‐based protein expression profiles in normal tissue, cancer and cell lines. IHC images of DHCR7 protein expression in clinical specimens of patients with LUAD and para carcinoma tissues were obtained from the Human Protein Atlas database.

### 2.4 Kaplan–Meier Plotter

The Kaplan–Meier Plotter tool (www.kmplot.com) includes survival information of 866 patients with LUAD. The prognostic value of DHCR7 expression was assessed by overall survival (OS), progression‐free survival (PFS) and post‐progression survival (PPS), using the hazard ratio (HR), 95% confidence intervals (CI) and log‐rank *p*‐value. In the analysis, patient samples were split into high expression group and low expression group based on the median mRNA levels of the DHCR7. The prognostic value of a gene was assessed by univariate Cox regression analysis. JetSet scores were used to select a single representative probe set for each gene. In the current study, only the probe sets with best JetSet scores for DHCR7 were selected to produce Kaplan–Meier plots. The one‐to‐one matches between DHCR7 genes and probe sets, identified by Affymetrix IDs, were as follows: DHCR72 and 272107_s_at; DHCR73 and 201555_at; DHCR74 and 222036_s_at; DHCR75 and 216237_s_at; DHCR76 and 238977_at; DHCR77 and 208795_s_at; DHCR78 and 224320_s_at; and DHCR710 and 223570_at. The relevant concepts are defined as follows: OS, time from diagnosis to death; PFS, time from diagnosis to tumor progression; PPS, time from progression to death; HR > 1, worse survival prognosis for the group with high mRNA expression; HR < 1, unfavorable survival prognosis in the low mRNA expression group; 95% CI does not cross 1, mRNA expression is associated with survival rate. As not all gene expression levels were available in all patients and only the JetSet probes were included in the study, the sample sizes vary for each survival analysis.

### 2.5 cBioPortal For Cancer Genomics Dataset

cBioPortal (http://cbioportal.org) is based on other authoritative databases, including the Gene Expression Omnibus (GEO; http://www.ncbi.nlm.nih.gov/geo/) and TCGA database. cBioPortal is a web resource for exploring, visualizing and analyzing multidimensional cancer genomics data. The genomic profile of each gene includes mutations, putative copy‐number alterations and mRNA expression *z*‐scores. The *z* score for each gene is the normalized expression of mRNA using the RNA‐Seq by expectation maximization count estimates method. The co‐expression of each gene pair was performed by Fisher's exact test and the network was constructed according to the correlation.

### 2.6 TISIDB Database for Relationship Between DHCR7 and Tumor Immune System

The TISIDB (http://cis.hku.hk/TISIDB/) is a web portal for tumor and immune system interaction, which integrates multiple heterogeneous data types. In this study, we analyzed the associations between DHCR7 expression and immune subtypes and molecular subtype across human cancers. The relationship between DHCR7 expression and pan‐cancer stages was also analyzed by TISIDB [13].

### 2.7 Ualcan Database

UALCAN (https://ualcan.path.uab.edu/index.html) [14] is a comprehensive, user‐friendly, and interactive web resource for analyzing cancer OMICS data. Here, we analyzed the staining of IHC and HE for DHCR7 in BLCA. The relationships between the differential DHCR7 expression in bladder cancer and clinical subgroups were analyzed by the UALCAN database.

### 2.8 LinkedOmics Database for DHCR7 co‐Expression Networks

The LinkedOmics database (http://www.linkedomics.org/login.php) is a powerful visual platform that facilitates the exploration of gene expression profiles [15]. In our study, we utilized LinkedOmics to identify the co‐expression genes of DHCR7 via Pearson's correlation coefficient, and visualized the results through heat maps and volcano plots. Subsequently, we employed gene set enrichment analysis (GSEA) to investigate the Gene Ontology biological processes (GO_BP) and KEGG pathways associated with DHCR7 and its co‐expression genes.

### 2.9 Cell Lines and Culture

The human renal cell carcinoma cell lines OSRC2, 786‐O, ACHN and the normal renal epithelial cell line NK2 were gifts from the Department of Laboratory Medicine, The First Affiliated Hospital of USTC, Division of Life Science and Medicine, University of Science and Technology of China, which were cultured in DMEM (GIBCO, Bethesda, MD, USA). All medium contained 10% fetal bovine serum (FBS), 100 μg/mL streptomycin and 100 units/mL penicillin and cells were maintained at 37°C, 5% CO_2_ in a humidified incubator. Cell lines were routinely tested for mycoplasma. Experiments were performed with noncontaminated cells.

### 2.10 Western Blotting

For western blotting, the treated cells were collected and washed with cold PBS, then the cells were lysed in protein extraction reagent (78 501, Thermo Scientific) containing protease and phosphatase inhibitor (1861281, Thermo Scientific). Protein concentrations were determined using a BCA Protein Assay Kit (Pierce), following the manufacturer's protocol. Equal amounts of protein (20 μg) were resolved by SDS‐PAGE on 10% polyacrylamide gels under reducing conditions. Subsequently, proteins were transferred onto PVDF membranes (Millipore) using a wet transfer system (Bio‐Rad) at 250 mA for 90 min. The bound antibodies were visualized using Super Signal reagents (Thermo Fisher Scientific). The mainly used primary antibodies in this study were DHCR7(Huabio.ER1907‐39) and GAPDH (Proteintech,60004‐1‐Ig). The horseradish peroxidase (HRP)‐conjugated secondary antibodies were acquired from Huabio.

### 2.11 shRNA Design and Lentiviral Vector Construction

Two shRNA sequences targeting human DHCR7 (NCBI Gene ID: 1717) were designed (BLOCK‐iT RNAi Designer, Thermo Fisher) and synthesized, with a scrambled shRNA (shCtrl) as a control. The sequences were cloned into pLKO.1‐puro (Addgene #8453), and lentiviruses were packaged in HEK293T cells using psPAX2/pMD2.G and Lipofectamine 3000. Supernatants were collected at 48/72 h, filtered (0.45 μm), and concentrated by ultracentrifugation (25 000 × g, 2 h). Target cells were transduced (MOI 5–10, 8 μg/mL polybrene) and selected with puromycin (2 μg/mL, 72 h). Stable pools were maintained in 1 μg/mL puromycin.

### 2.12 Cell Proliferation Assays

For cell proliferation assay, the treated cells were labeled by incubating with 10 μM EdU (5‐ethynyl‐2ˊ‐deoxyuridine) for 2 h at 37°C. After fixation with 4% paraformaldehyde (PFA) for 15 min, cells were permeabilized with 0.3% Triton X‐100 in PBS for 10 min. The BeyoClick EdU‐DAB Kit (Beyotime, Cat# C0085S) was used for detection. Cells were incubated with the Click Reaction Mix (containing biotin‐azide and Cu^2+^ catalyst) for 30 min at room temperature, protected from light. After washing, samples were treated with horseradish peroxidase (HRP)‐streptavidin for 30 min, followed by DAB (3,3ˊ‐diaminobenzidine) substrate to develop a brown precipitate. Nuclei were counterstained with hematoxylin. Proliferating (EdU‐positive) cells were quantified under a light microscope.

## 3 Results

### 3.1 The Expression of DHCR7 Gene in Human Pan‐Cancer

**FIGURE 1:**
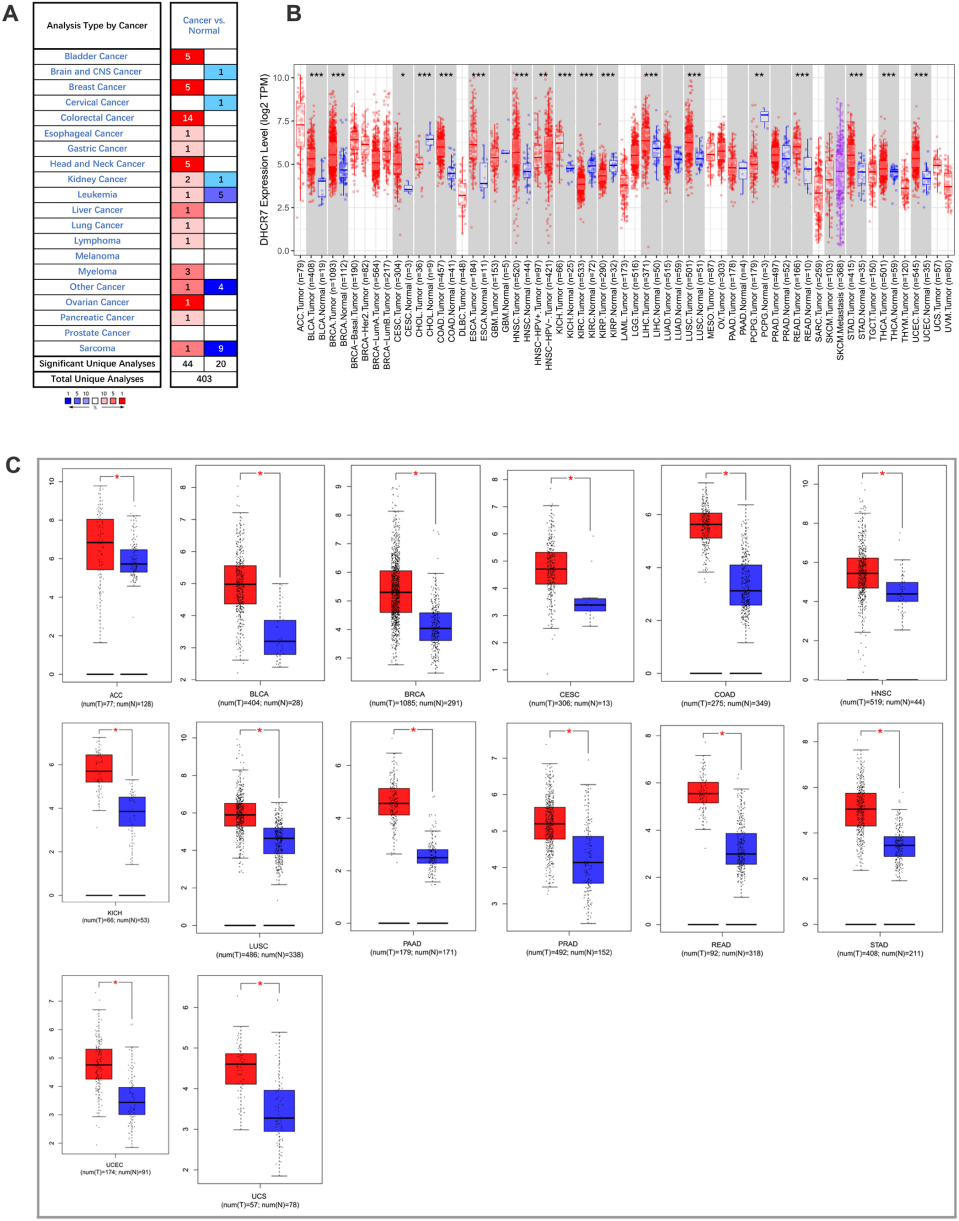
DHCR7 expression levels in human cancers. (A) DHCR7 expression in different cancers and paired normal tissue in the Oncomine database. (B) DHCR7 expression levels in different cancer types from the TCGA database analyzed by the TIMER database. (C) DHCR7 expression in several cancers and paired normal tissue in the GEPIA database (*p < 0.05, **p < 0.01, ***p < 0.001).

The Oncomine database demonstrated that mRNA expression of DHCR7 was significantly higher in various human cancers, including colorectal cancer, bladder cancer, breast cancer, head and neck cancer, and ovarian cancer, when compared to corresponding normal tissues. However, DHCR7 expression was found to be significantly lower in brain and CNS cancer, cervical cancer, melanoma, and prostate cancer (Figure 1A). Furthermore, the TIMER database revealed that DHCR7 expression was significantly higher in several cancer types, such as BLCA (bladder urothelial carcinoma), BRCA (breast invasive carcinoma), CESC (cervical squamous cell carcinoma and endocervical adenocarcinoma), COAD (colon adenocarcinoma), ESCA (esophageal carcinoma), HNSC (head and neck cancer), KICH (kidney chromophobe), LIHC (liver hepatocellular carcinoma), LUAD (lung adenocarcinoma), LUSC (lung squamous cell carcinoma), READ (rectum adenocarcinoma), STAD (stomach adenocarcinoma), THCA (thyroid carcinoma), and UCEC (uterine corpus endometrial carcinoma). Conversely, DHCR7 mRNA expression was low in CHOL (cholangiocarcinoma), KIRC (kidney renal clear cell carcinoma), KIRP (kidney renal papillary carcinoma), and PCPG (pheochromocytoma and paraganglioma) (Figure 1B). Consistently, the GEPIA database displayed significant elevation of DHCR7 mRNA expression across most cancer types (Figure 1C). Additionally, the UALCAN database analysis indicated significant differential expression of DHCR7 between tumors and healthy tissues in various human cancers (Figure S1). Overall, these results suggested that DHCR7 is overexpressed in most human cancer tissues.

### 3.2 Highly Expressed DHCR7 Is Associated With Poor Prognosis in Human Cancers

**FIGURE 2:**
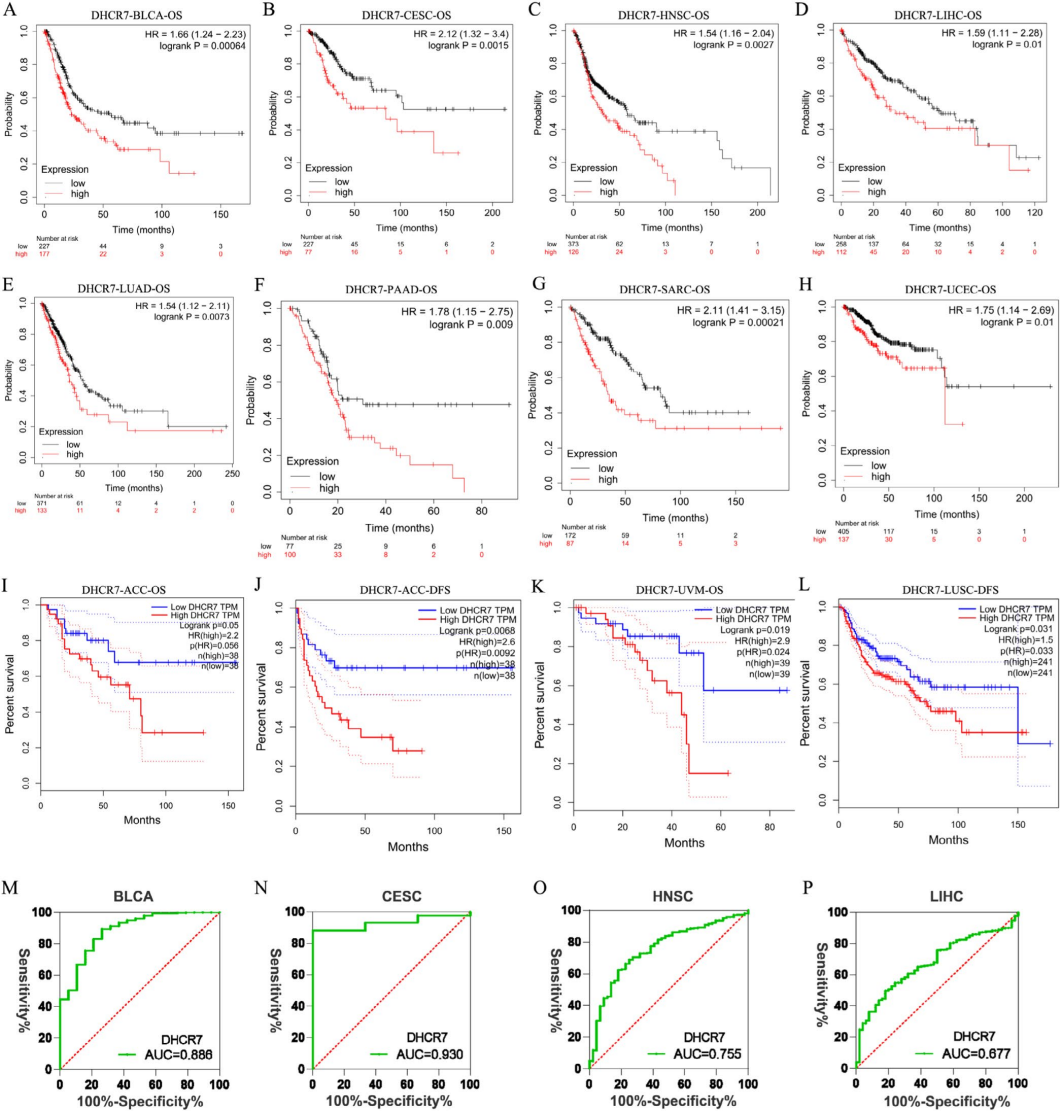
Highly DHCR7 expression is associated with poor overall survival in human cancers. Kaplan–Meier survival curve of human cancers with high and low DHCR7 expression analyzed by the Kaplan–Meier plotter database (A–H) and the GEPAI database (I–L). (A–H) Highly DHCR7 expression was related to worse OS in BLCA (*n* = 404), CESC(*n* = 304), HNSC(*n* = 499), LIHC(*n* = 370), LUAD(*n* = 504), PAAD(*n* = 177), SARC(*n* = 259), and UCEC(*n* = 542) cohorts. (I–J) High DHCR7 expression was related to worse OS and DFS in ACC (*n* = 76) cohorts. (K) High DHCR7 expression was related to worse OS in UVM (*n* = 78) cohorts. (L) High DHCR7 expression was related to worse DFS in LUSC cohorts (*n* = 482). (M–P) ROC curve analysis of DHCR7 expression in BLCA, CESC, HNSC, and LIHC. DFS, disease‐free survival; OS, overall survival; ROC, receiver operating characteristic curve.

Given the high expression of DHCR7 in many cancer cells, it is important to examine its relationship with OS or disease‐free survival (DFS). We then conducted an analysis using multiple databases. Firstly, from the Kaplan–Meier plotter database, we found that higher DHCR7 expression was associated with poor OS in BLCA (*n* = 404, HR = 1.66, *p* = 0.00064) (Figure 2A), CESC (*n* = 304, HR = 2.12, *p* = 0.0015) (Figure 2B), HNSC (*n* = 499, HR = 1.54, *p* = 0.0027) (Figure 2C), LIHC (*n* = 370, HR = 1.59, *p* = 0.01) (Figure 2D), LUAD(*n* = 504, HR = 1.54, *p* = 0.0073) (Figure 2E), PAAD(*n* = 177, HR = 1.78, *p* = 0.009) (Figure 2F), SARC(*n* = 259, HR = 2.11, *p* = 0.00021) (Figure 2G) and UCEC(*n* = 542, HR = 1.75, *p* = 0.01) (Figure 2H). Secondly, a higher DHCR7 expression was related to worse OS (*n* = 76, HR = 2.2, *p* = 0.05) (Figure 2I) and disease‐free survival (DFS) (*n* = 76, HR = 2.6, *p* = 0.0068) (Figure 2J) in ACC by GEPIA database. We also found that higher DHCR7 expression was related to worse OS in UVM (*n* = 78, HR = 2.9, *p* = 0.019) (Figure 2K). A higher DHCR7 expression was related to worse DFS in LUSC (*n* = 482, HR = 1.5, *p* = 0.031) (Figure 2L). Similarly, we found that higher DHCR7 expression was related to poor prognosis in human cancers from the TISIDB database (Figure S2). To further evaluate the diagnostic potential of DHCR7 expression in cancer, we performed receiver operating characteristic (ROC) curve analysis in BLCA, CESC, HNSC, and LIHC. The area under the ROC curve (AUC) values for DHCR7 were 0.886 (BLCA), 0.930 (CESC), 0.755 (HNSC), and 0.677 (LIHC), respectively. The 95% confidence intervals for BLCA, CESC, HNSC, LIHC were 0.8091–0.9619, 0.8790–0.9806, 0.6855–0.8250, and 0.6093–0.7440 (Figure 2M–P). Collectively, these results indicate that highly expressed DHCR7 is associated with poor prognosis and might be a potential diagnostic biomarker in most human cancers.

### 3.3 DHCR7 Expression Correlates With Pathological Staging and Metastatic Progression in Human Cancers

**FIGURE 3:**
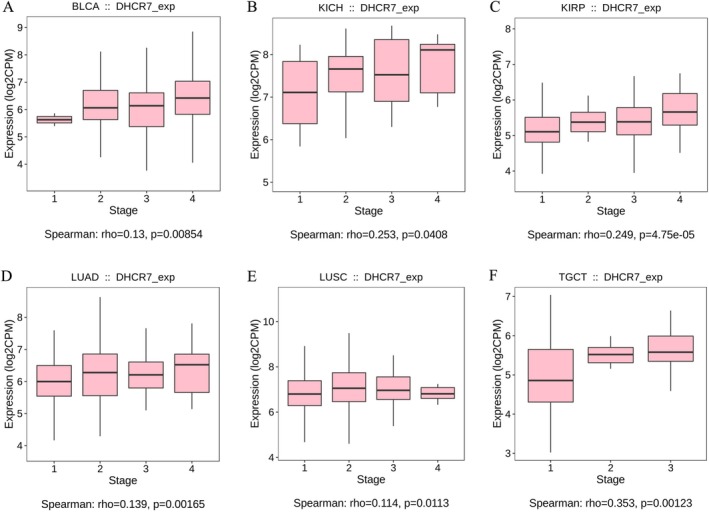
The relationship between DHCR7 expression and pan‐cancer stages by TISIDB. (A) in BLCA, (B) in KICH, (C) in KIRP, (D) in LUAD, (E) in LUSC, (F) in TGCT.

**FIGURE 4:**
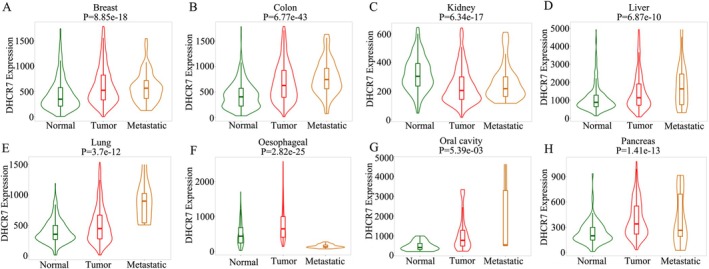
The relationship between DHCR7 expression and pan‐cancer metastasis. (A) in Breast, (B) in Colon, (C) in Kidney, (D) in Liver, (E) in Lung, (F) in Oesophageal, (G) in Oral cavity, (H) in Pancreas.

Then, we investigate the association between DHCR7 expression and tumor pathological staging as well as metastasis. The TISIDB database showed that the mRNA levels of DHCR7 were significantly differentially expressed between the tumor stages I and III or IV in BLCA (spearman *r* = 0.13, *p* = 0.00854) (Figure 3A), KICH (spearman *r* = 0.253, *p* = 0.0408) (Figure 3B), KIRP (spearman *r* = 0.249, *p* = 4.75e‐05) (Figure 3C), LUAD (spearman *r* = 0.139, *p* = 0.00165) (Figure 3D), LUSC (spearman *r* = 0.114, *p* = 0.0113) (Figure 3E) and TGCT (spearman *r* = 0.353, *p* = 0.00123) (Figure 3F). The Oncomine database also showed that DHCR7 was highly expressed in BLCA (Figure S3). In addition, we analyzed the differential expression of DHCR7 in tumor, normal, and metastatic tissues with TNMplot.com [16]. According to the gene chip data from the TNM‐plot, we can see that DHCR7 is differently expressed in breast invasive carcinoma (p(Kruskal‐Wallis) = 8.85e‐18, n(normal) = 242, n(tumor) = 7569 and n(metastatic) = 82) (Figure 4A), colon cancer (p(Kruskal‐Wallis) = 6.77e‐43, n(normal) = 377, n(tumor) = 1450 and n(metastatic) = 99) (Figure 4B), kidney cancer (p(Kruskal‐Wallis) = 6.34e‐17, n(normal) = 277, n(tumor) = 556 and n(metastatic) = 58) (Figure 4C), hepatic carcinoma(p(Kruskal‐Wallis) = 6.87e‐10, n(normal) = 379, n(tumor) = 806 and n(metastatic) = 24) (Figure 4D), lung cancer (p(Kruskal‐Wallis) = 3.7e‐12, n(normal) = 391, n(tumor) = 1865 and n(metastatic) = 8) (Figure 4E), oesophageal carcinoma (p(Kruskal‐Wallis) = 2.82e‐25, n(normal) = 88, n(tumor) = 440 and n(metastatic) = 28) (Figure 4F), oral cancer (p(Kruskal‐Wallis) = 5.39e‐03, n(normal) = 18, n(tumor) = 38 and n(metastatic) = 5) (Figure 4G) and pancreatic cancer(p(Kruskal‐Wallis) = 1.41e‐13, n(normal) = 108, n(tumor) = 248 and n(metastatic) = 17) (Figure 4H). Based on the above results, elevated expression of DHCR7 was observed in advanced stages and metastatic tissues of human cancers, which means DHCR7 might be an optimal prognostic, tumor progression, and novel therapeutic biomarker.

### 3.4 DHCR7 Expression Is Associated With Immune and Molecular Subtypes of Human Cancers

**FIGURE 5:**
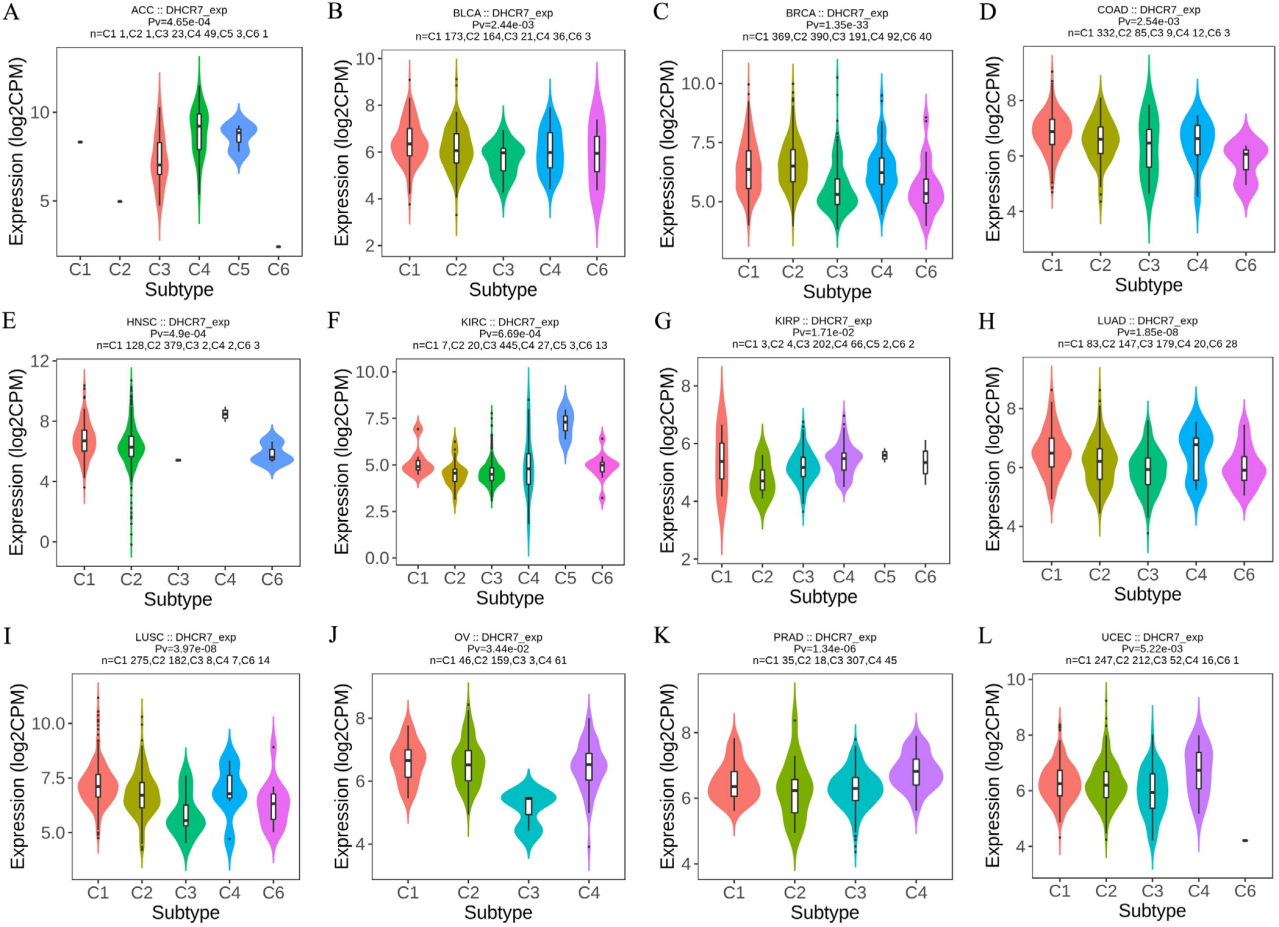
The relationship between DHCR7 expression and pan‐cancer immune subtypes. (A) in ACC, (B) in BLCA, (C) in BRCA, (D) in COAD, (E) in HNSC, (F) in KIRC, (G) in KIRP, (H) in LUAD, (I) in LUSC, (J) in OV, (K) in PRAD, (L) in UCEC.

**FIGURE 6:**
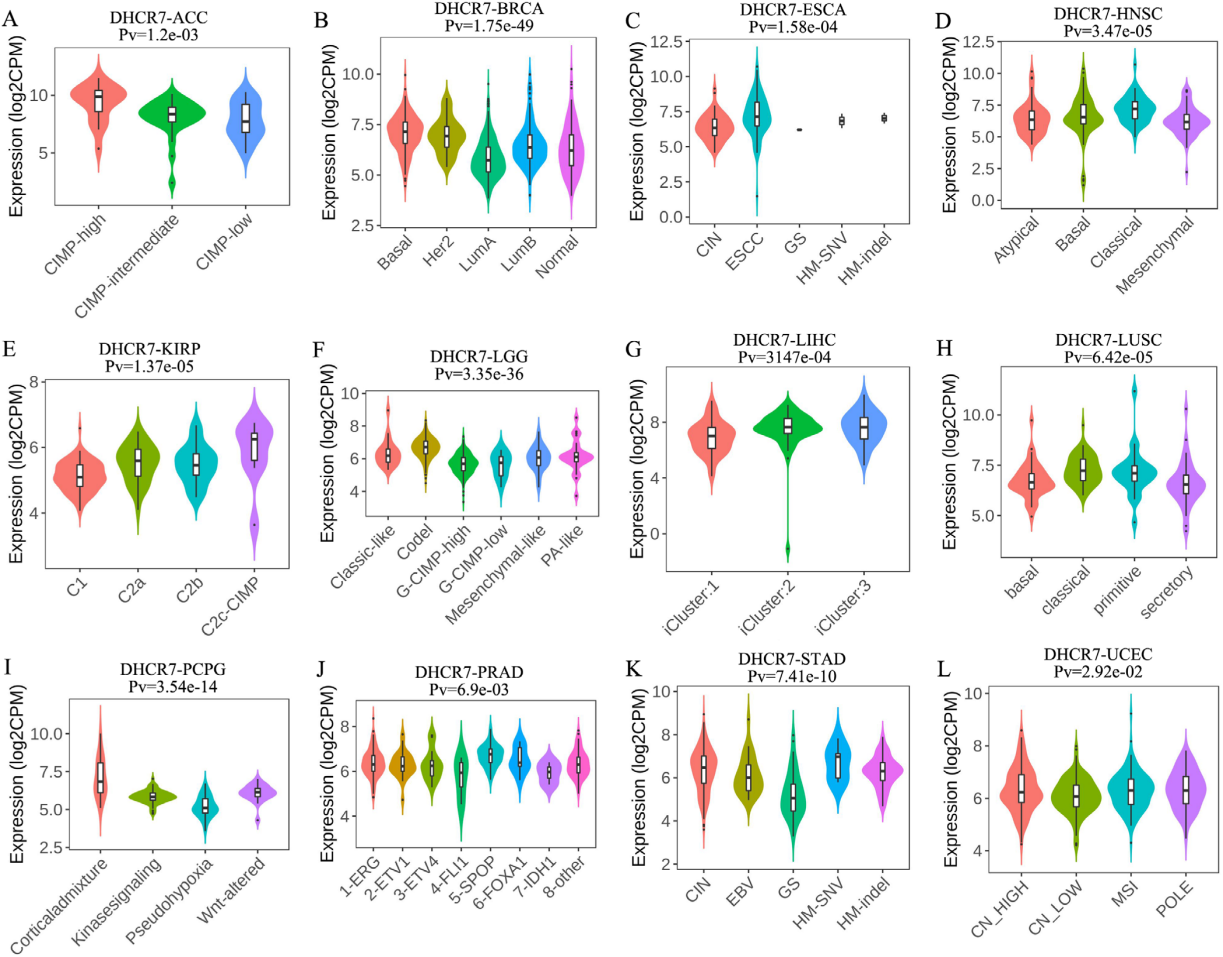
The relationship between DHCR7 expression and pan‐cancer molecular subtypes. (A) in ACC, (B) in BRCA, (C) in ESCA, (D) in HNSC, (E) in KIRP, (F) in LGG, (G) in LIHC, (H) in LUSC, (I) in PCPG, (J) in PRAD, (K) in STAD, (L) in UCEC.

We next explored the DHCR7 expression on immune and molecular subtypes among human cancers by the TISIDB database. Immune subtypes consist of six types, which were C1(wound healing), C2(IFN‐γ dominant), C3(inflammatory), C4 (lymphocyte depleted), C5 (immunologically quiet), and C6 (TGF‐β dominant). The results showed that DHCR7 expression was related to different immune subtypes in ACC, BLCA, BRCA, COAD, HNSC, KIRC, KIRP, LUAD, LUSC, OV, PRAD, and UCEC (Figure 5). Particularly, DHCR7 expression varied among different immune subtypes in various tumor types. For instance, DHCR7 exhibited high expression in C5 subtypes of ACC and KIRC. However, in C3 subtypes of LUAD, OV, BRCA, and LUSC, DHCR7 displayed low expression. Regarding molecular subtypes of cancers, our analysis revealed a significant association between DHCR7 expression and ACC, BRCA, ESCA, HNSC, KIRP, LGG, LIHC, LUSC, PCPG, PRAD, STAD, and UCEC (Figure 6). These findings demonstrate that DHCR7 expression varies across immune subtypes and molecular subtypes in various human tumors.

### 3.5 DHCR7 Expression and Immune Checkpoint (ICP) Genes in Pan Cancers

**FIGURE 7:**
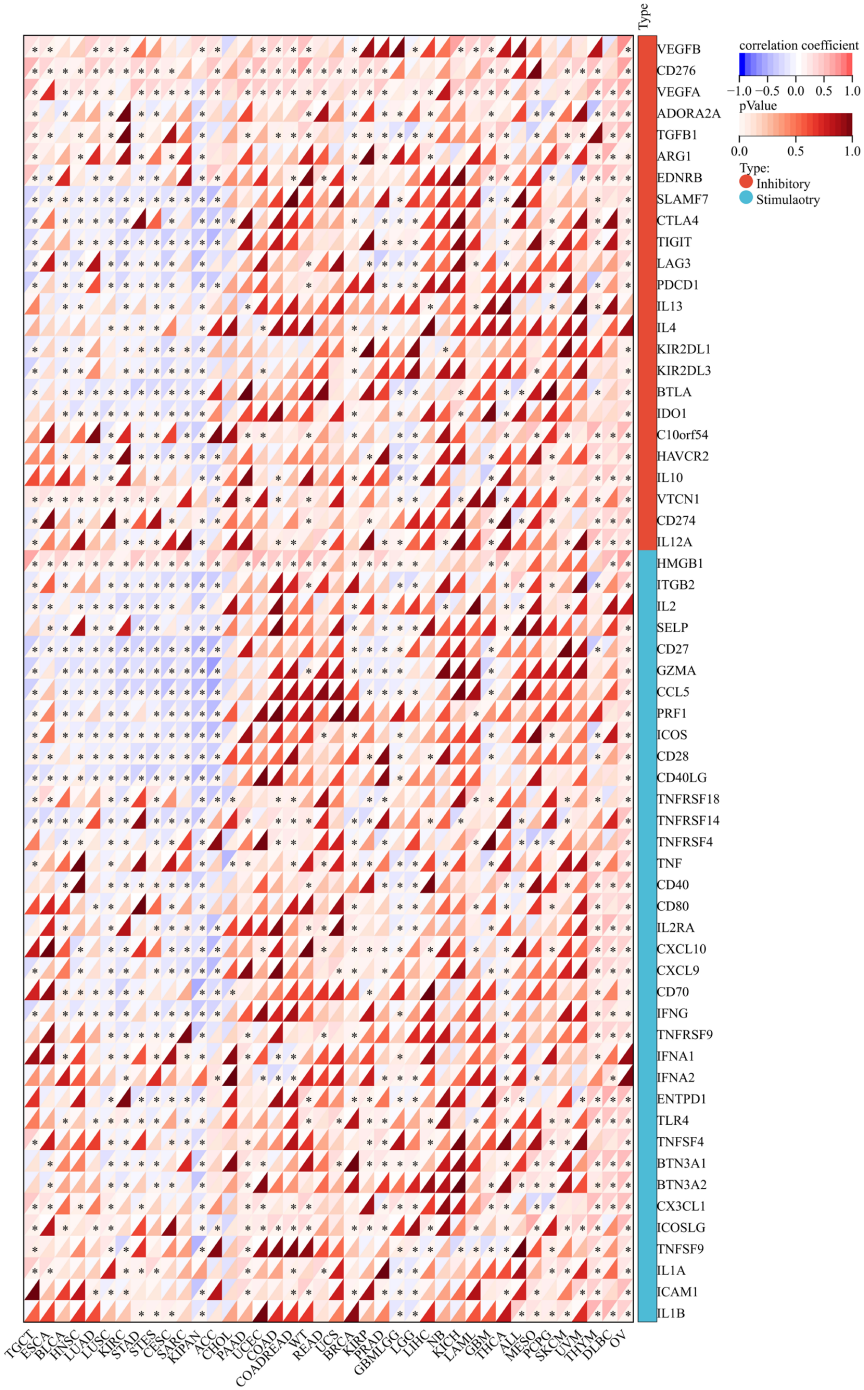
The relationship between DHCR7 expression and pan‐cancer immune checkpoint genes. *p < 0.05; **p < 0.01; ***p < 0.001.

Immune checkpoint (ICP) inhibitors targeting CTLA‐4, PD‐1, and PD‐L1 have shown significant clinical efficacy in treating various cancers, including lung cancer, melanoma, and bladder cancer. However, a considerable proportion of patients remain refractory to these therapies, highlighting the urgent need to elucidate the underlying resistance mechanisms [17, 18]. Therefore, we analyzed the relationship between DHCR7 expression and ICP in the TEM. Among 60 ICP genes, including 24 immune inhibitors (such as CTLA4, LAG3, or VEGFB) and 36 activators (TNFRSF9, CD28, or TNFRSF9, etc.), we found DHCR7 played roles both in inhibitors and activators of ICP in human cancers (Figure 7). For example, positive correlations between DHCR7 expression and the immune inhibitors CD276 and VEGFA were found in many cancers, such as TGCT, BLCA, HNSC, LUAD, LUSC, STAD, STES, CESC, SARC, and PRAD, etc. For immune activators, negative correlations between DHCR7 expression and CD27, GZMA, CCL5, PRF1, ICOS, CD28, or CD40LG were shown in BLCA, HNSC, LUAD, LUSC, KIRC, STAD, STES, CESC, SARC, KIPAN, and ACC. These findings strongly support DHCR7 as a promising pan‐cancer biomarker and a potential therapeutic target for immunotherapy.

### 3.6 DHCR7 Expression Is Related to Microsatellite Instability (MSI), neoantigen, Tumor Mutational Burden (TMB), and Mutant‐Allele Tumor Heterogeneity (MATH)

**FIGURE 8:**
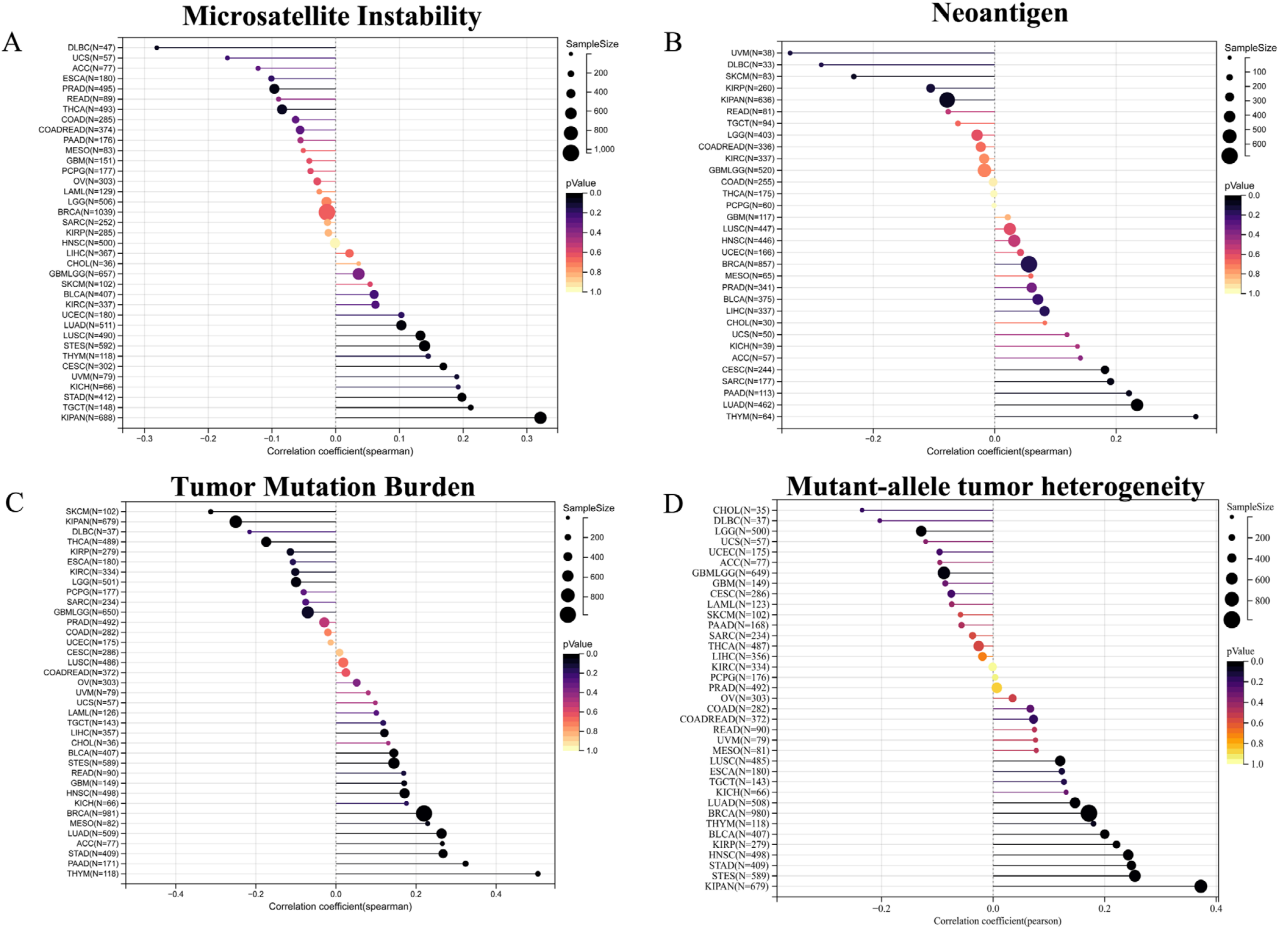
The relationship between DHCR7 expression and MSI (A), Neoantigen (B), TMB (C) and MATH (D) in human cancers. MATH, mutant‐allele tumor heterogeneity; MSI, microsatellite instability; TMB, tumor mutational burden.

Multiple studies have shown that microsatellite instability, neoantigen burden, tumor mutational burden, and mutant‐allele tumor heterogeneity are strongly associated with immunotherapy response [19, 20, 21, 22]. To characterize the roles of DHCR7 in the immune response of the TME, we analyzed the correlations between the DHCR7 expression and TMB, MSI, neoantigen and mutant allele tumor heterogeneity (MATH). We observed that DHCR7 had significant positive correlations with MSI in 4 tumors: CESC (*N* = 302, *R* = 0.127, *p* = 0.028), LUAD (*N* = 511, *R* = 0.122, *p* = 0.006), KIPAN (*N* = 688, *R* = 0.341, *p* = 3.50e‐20), TGCT (*N* = 148, *R* = 0.237, *p* = 0.004), and negative relation in 3 tumors: PRAD (*N* = 495, *R* = −0.120, *p* = 0.008), THCA (*N* = 493, *R* = −0.094, *p* = 0.036), UCS (*N* = 57 *R* = −0.274, *p* = 0.039) (Figure 8A). For neoantigen, we observed DHCR7 had a significant positive correlation in LUAD (*N* = 462, *R* = 0.165, *p* = 0.001) (Figure 8B). Analyzing the relationships between DHCR7 expression and TMB, we found that DHCR7 expression was positively related to TMB in GBM(*N* = 149, *R* = 0.170, *p* = 0.038), LUAD(*N* = 509, *R* = 0.264, *p* = 1.576e‐9), BRCA(*N* = 981, *R* = 0.220, *p* = 3.339e‐12), STES(*N* = 589, *R* = 0.145, *p* = 0.0004), STAD(*N* = 409, *R* = 0.268, *p* = 3.934e‐8), HNSC(*N* = 498, *R* = 0.171, *p* = 0.0001), THYM(*N* = 118, *R* = 0.504, *p* = 5.781e‐9), LIHC(*N* = 357, *R* = 0.121, *p* = 0.022), MESO(*N* = 82, *R* = 0.229, *p* = 0.040), PAAD(*N* = 171, *R* = 0.324, *p* = 0.00001), BLCA(*N* = 407, *R* = 0.145, *p* = 0.003), ACC(*N* = 77, *R* = 0.266, *p* = 0.019) and negatively related to TMB in LGG(*N* = 501, *R* = −0.101, *p* = 0.025), KIPAN(*N* = 679, *R* = −0.250, *p* = 3.757e‐11), THCA(*N* = 489, *R* = −0.174, *p* = 0.0001), and SKCM(*N* = 102, *R* = −0.313, *p* = 0.001) (Figure 8C). We also found that DHCR7 expression was positively related to MATH in LUAD(*N* = 508, *R* = 0.163, *p* = 0.0002), BRCA(*N* = 980, *R* = 0.198, *p* = 4.193e‐10), STES(*N* = 589, *R* = 0.281, *p* = 3.618e‐12), KIPAN(*N* = 679, *R* = 0.166, *p* = 0.00001), STAD(*N* = 409, *R* = 0.250, *p* = 3.026e‐7), HNSC(*N* = 498, *R* = 0.237, *p* = 9.016e‐8), LUSC(*N* = 485, *R* = 0.102, *p* = 0.025), THYM(*N* = 118, *R* = 0.185, *p* = 0.044), and BLCA(*N* = 407, *R* = 0.198, *p* = 0.00005) and negatively related to MATH in GBMLGG(*N* = 649, *R* = −0.097, *p* = 0.014), LGG(*N* = 500, *R* = −0.130, *p* = 0.003) and DLBC(*N* = 37, *R* = −0.349, *p* = 0.034) (Figure 8D). These results provide further evidence to suggest that the DHCR7 gene may play a critical role in the immunotherapy of human tumors.

### 3.7 Relationship Between DHCR7 Expression and Immune Cell Infiltration in Pan‐Cancer

**FIGURE 9:**
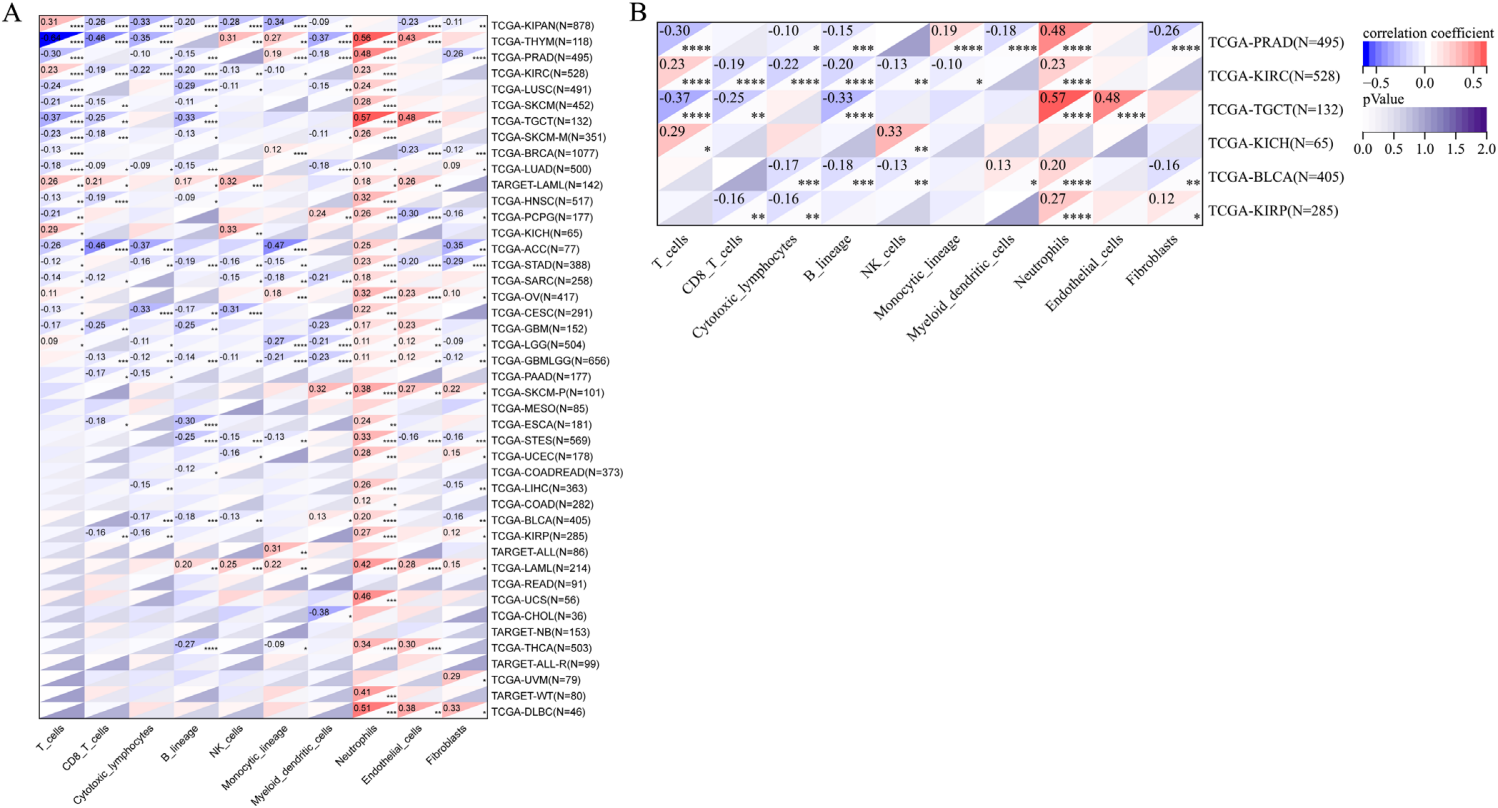
The relationship between DHCR7 expression and infiltrating immune cells of human cancers and urogenital cancers. (A) The relationship between DHCR7 expression level and infiltrating levels of B cells, CD4+ T cells, CD8+ T cells, macrophages, neutrophils, dendritic cells in human cancers. (B) The relationship between DHCR7 expression level and infiltrating levels of B cell lineages, CD8+ T cells, cytotoxic lymphocytes, endothelial cells, fibroblasts, monocytic cell lineages, myeloid dendritic cells, neutrophils, natural killer cells, T cells in six urogenital cancers. *p < 0.05; **p < 0.01; ***p < 0.001.

The presence of multiple immune cell infiltrations in the tumor immune microenvironment can influence tumorigenesis, progression and even metastasis. Therefore, we analyzed the potential connection of DHCR7 expression and immune cell infiltration in the TME of human cancers. We obtained 10 categories of immune cell infiltration scores for 10 180 tumor samples in a total of 44 tumor types. We calculated the Pearson's correlation coefficient between gene and immune cell infiltration scores in each tumor using the corr.test function of the R package psych (version 2.1.6) to identify significantly correlated immune infiltration scores, and finally we observed that in 40 cancer species (TCGA‐GBM (*N* = 152), TCGA‐GBMLGG (*N* = 656), TCGA‐LGG (*N* = 504), TCGA‐UCEC (*N* = 178), TARGET‐LAML (*N* = 142), TCGA‐BRCA (*N* = 1077), TCGA‐CESC (*N* = 291), TCGA‐LUAD (*N* = 500), TCGA‐ESCA (*N* = 181), TCGA‐STES (*N* = 569), TCGA‐SARC (*N* = 258), TCGA‐KIRP (*N* = 285), TCGA‐KIPAN (*N* = 878), TCGA‐COAD (*N* = 282), TCGA‐COADREAD (*N* = 373), TCGA‐PRAD (*N* = 495), TCGA‐STAD (*N* = 388), TCGA‐HNSC (*N* = 517), TCGA‐KIRC (*N* = 528), TCGA‐LUSC (*N* = 491), TCGA‐THYM (*N* = 118), TCGA‐LIHC (*N* = 363), TARGET‐WT (*N* = 80), TCGA‐SKCM‐P (*N* = 101), TCGA‐SKCM (*N* = 452), TCGA‐BLCA (*N* = 405), TCGA‐SKCM‐M (*N* = 351), TCGA‐THCA (*N* = 503), TCGA‐OV (*N* = 417), TCGA‐UVM (*N* = 79), TCGA‐PAAD (*N* = 177), TCGA‐TGCT (*N* = 132), TCGA‐UCS (*N* = 56), TCGA‐LAML (*N* = 214), TARGET‐ALL (*N* = 86), TCGA‐PCPG (*N* = 177), TCGA‐ACC (*N* = 77), TCGA‐DLBC (*N* = 46), TCGA‐KICH (*N* = 65), TCGA‐CHOL (*N* = 36)) in which the gene expression was significantly associated with immune in cells filtration (Figure 9A). The results demonstrated a robust correlation between DHCR7 expression and various immune cell types in human cancers, including T‐cells, CD8 T‐cells, cytotoxic lymphocytes, B‐lineage cells, NK‐cells, monocytic lineage cells, myeloid dendritic cells, neutrophils, endothelial cells, and fibroblasts. These findings were consistent across six urogenital cancers (Figure 9B). Additionally, using the TIMER database, we analyzed the correlation between DHCR7 and immune cell‐related genes and markers. Table 1 illustrates that DHCR7 displayed close connections with all included marker genes of CD8+ T cells, monocytes, dendritic cells, regulatory T cells (Tregs), and T cell exhaustion markers in BLCA, KIRC, and PRAD. Notably, there was no correlation between gene markers of M1 macrophages and DHCR7 expression. However, DHCR7 expression showed moderate to strong correlations with M2 macrophage markers, such as CD163, VSIG4, and MS4A4A in BLCA. Furthermore, DHCR7 expression exhibited positive effects on the expression of Treg and T cell exhaustion markers, including FOXP3, STAT5B, TGFB1, PDCD1, CTLA4, and LAG3 in BLCA, KIRC, and PRAD. Additionally, DHCR7 expression demonstrated significant correlations with the regulation of several markers of T helper cells (Th1, Th2, Tfh, and Th17) in the four bladder urothelial carcinomas. In summary, our findings establish DHCR7 as a key regulator of immune cell recruitment and function in the TEM across multiple cancer types. Importantly, DHCR7 demonstrates strong potential as a prognostic biomarker, particularly in bladder urothelial carcinoma, with broader implications for other malignancies.

**TABLE 1:** Correlation between DHCR7 and related genes and markers of immune cells analyzed by TIMER.

Immune cells	Gene Markers	BLCA				KIRC				PRAD			
		None		Tumor purity		None		Tumor purity		None		Tumor purity	
		cor	*p*	cor	*p*	cor	*p*	cor	*p*	cor	*p*	cor	*p*
CD8 + T cell	CD8A	−0.19586	***	−0.2236	***	−0.27577	***	−0.27335	***	−0.12146	**	−0.11132	*
	CD8B	−0.11777	**	−0.14657	**	−0.2787	***	−0.27239	***	−0.10822	*	−0.12419	*
T cell (general)	CD3D	−0.32385	***	−0.36339	***	−0.33614	***	−0.3344	***	−0.27501	***	−0.27543	***
	CD3E	−0.27328	***	−0.30403	***	−0.31097	***	−0.30536	***	−0.19392	***	−0.19211	***
	CD2	−0.27943	***	−0.31199	***	−0.32859	***	−0.32213	***	−0.19479	***	−0.1946	***
B cell	CD19	−0.05365	0.279619	−0.07139	0.171163	−0.24673	***	−0.27105	***	−0.20878	***	−0.2065	***
	CD79A	−0.12671	**	−0.1645	**	−0.19528	***	−0.21836	***	−0.18398	***	−0.17851	***
Monocyte	CD86	−0.18119	**	−0.20862	***	−0.19211	***	−0.20086	***	−0.10715	*	−0.11134	*
	CD115	−0.23229	***	−0.26768	***	−0.11285	**	−0.12108	**	−0.11621	**	−0.12571	*
TAM	CCL2	−0.0876	0.07717	−0.10832	*	−0.10188	*	−0.05923	0.203781	−0.18444	***	−0.17476	***
	CD68	−0.16595	***	−0.18553	***	−0.05774	0.183156	−0.08528	0.067035	−0.03301	0.462155	−0.0363	0.459583
	IL10	−0.11568	*	−0.13589	**	−0.08411	0.052303	−0.09249	*	−0.0611	0.173382	−0.07017	0.152592
Macrophage‐M1	NOS2	−0.00274	0.955981	−0.02286	0.66162	0.081295	0.060718	0.10275	*	−0.02164	0.63005	−0.02173	0.658219
	IRF5	0.096087	0.05248	0.111088	*	−0.17498	***	−0.15033	**	0.072285	0.107126	0.103115	*
	PTGS2	0.011213	0.821281	0.001386	0.978831	0.087768	*	0.091998	*	0.060041	0.180995	0.054614	0.265828
Macrophage‐M2	CD163	−0.15245	**	−0.1891	***	−0.01901	0.661493	−0.05537	0.234928	0.007629	0.865096	0.00037	0.993996
	VSIG4	−0.1864	***	−0.22247	***	−0.08084	0.062174	−0.10739	*	−0.03484	0.437809	−0.03774	0.441989
	MS4A4A	−0.18064	***	−0.2142	***	−0.13268	**	−0.16096	***	−0.08323	0.063493	−0.07633	0.119588
Neutrophils	CEACAM8	−0.00431	0.930789	0.011544	0.825081	−0.03744	0.38828	−0.04199	0.367915	0.051811	0.24847	0.101321	*
	ITGAM	−0.12712	*	−0.15503	**	−0.06124	0.157988	−0.07227	0.120854	−0.09804	*	−0.09619	*
	CCR7	−0.02074	0.676044	−0.03918	0.452835	−0.16285	***	−0.16061	***	−0.16081	***	−0.15697	**
Natural killer cell	KIR2DL1	−0.14443	**	−0.14914	**	−0.13333	**	−0.14512	**	0.039004	0.385089	0.050647	0.302164
	KIR2DL3	−0.18381	***	−0.21371	***	−0.16468	**	−0.18969	***	0.020434	0.649179	0.028821	0.557277
	KIR2DL4	−0.13726	**	−0.15328	**	−0.22226	***	−0.24726	***	0.017534	0.696292	0.055672	0.256662
	KIR3DL1	−0.16485	***	−0.19897	***	−0.0978	*	−0.10347	**	0.038837	0.387129	0.043741	0.372946
	KIR3DL2	−0.16592	***	−0.18724	***	−0.19947	***	−0.17963	***	−0.02281	0.611616	−0.0341	0.487344
	KIR3DL3	−0.09963	*	−0.10519	*	−0.01217	0.779233	−0.0012	0.979446	−0.02108	0.638803	−0.07077	0.149136
	KIR2DS4	−0.13573	**	−0.13453	**	−0.08565	*	−0.08946	0.054668	−0.06541	0.14493	−0.0661	0.177933
Dendritic cell	HLA‐DPB1	−0.23534	***	−0.26663	***	−0.19388	***	−0.20273	***	−0.24614	***	−0.23612	***
	HLA‐DQB1	−0.21543	***	−0.23665	***	−0.15934	***	−0.14109	**	−0.125	**	−0.14427	**
	HLA‐DRA	−0.19863	***	−0.22956	***	−0.17455	***	−0.19092	***	−0.11879	**	−0.1005	*
	HLA‐DPA1	−0.19589	***	−0.22564	***	−0.18214	***	−0.18518	***	−0.09946	*	−0.09499	0.052596
	CD1C	−0.19538	***	−0.20709	***	−0.06754	0.11937	−0.07661	0.100055	−0.13325	**	−0.15735	**
	NRP1	−0.06522	0.188496	−0.0713	0.171612	0.086322	*	0.112576	*	0.201506	***	0.192022	***
	ITGAX	−0.15404	**	−0.18832	***	−0.20435	***	−0.19743	***	−0.11349	*	−0.10169	*
Th1	TBX21	−0.23853	***	−0.27459	***	−0.26546	***	−0.25502	***	−0.16884	***	−0.1529	**
	STAT4	−0.24785	***	−0.26884	***	−0.3496	***	−0.31295	***	−0.21003	***	−0.21232	***
	STAT1	−0.03934	0.427908	−0.06087	0.243304	−0.06085	0.160673	−0.05711	0.22048	0.268138	***	0.287683	***
	IFNG	−0.15589	**	−0.17121	***	−0.30543	***	−0.3137	***	−0.08633	0.054205	−0.06347	0.19582
	TNF	−0.04022	0.417814	−0.06405	0.219679	−0.12719	**	−0.11341	*	−0.0975	*	−0.09202	0.060444
	IL12A	−0.08385	0.090752	−0.0957	0.066309	−0.17562	***	−0.18341	***	−0.22644	***	−0.24393	***
	IL12B	−0.13796	0.005247	−0.14836	**	−0.14222	***	−0.1657	***	−0.11381	*	−0.1028	*
Th2	GATA3	0.153558	**	0.181402	***	−0.07492	0.083976	−0.08182	0.078953	−0.11032	*	−0.11487	*
	STAT6	−0.06489	0.190742	−0.04912	0.3466	−0.01079	0.803703	−0.00335	0.942796	0.080051	0.074302	0.071302	0.146031
	STAT5A	−0.15018	**	−0.1785	***	−0.092	*	−0.08571	0.065684	−0.14754	***	−0.13677	**
	IL13	−0.1454	**	−0.13813	**	−0.29142	***	−0.26998	***	−0.10955	*	−0.07775	0.112881
Tfh	BCL6	0.153717	**	0.142101	**	−0.08989	**	−0.07397	0.112335	0.052537	0.241802	0.089981	0.066423
	IL21	−0.06827	0.16868	−0.06784	0.193508	−0.05836	0.178533	−0.07018	0.132023	0.000546	0.990302	−0.00513	0.916783
Th17	STAT3	0.069958	0.158339	0.049817	0.339768	0.19775	***	0.217759	***	0.402702	***	0.416261	***
	IL17A	−0.09549	0.053953	−0.09111	0.080475	−0.04758	0.272818	−0.02671	0.566868	−0.02434	0.587972	−0.02065	0.674128
Treg	FOXP3	−0.1487	**	−0.18077	***	−0.26485	***	−0.26934	***	0.057371	0.20121	0.071051	0.147511
	CCR8	−0.05056	0.308306	−0.07937	0.128035	−0.18279	***	−0.18708	***	0.020936	0.641163	0.033503	0.495061
	STAT5B	0.099503	*	0.084883	0.103514	0.206569	***	0.223052	***	0.101641	*	0.112516	*
	TGFB1	−0.15477	**	−0.15917	**	0.08664	*	0.105046	*	−0.15077	***	−0.12738	**
T cell exhaustion	PDCD1	−0.20637	***	−0.24028	***	−0.27119	***	−0.25947	***	−0.22522	***	−0.22395	***
	CTLA4	−0.21661	***	−0.25923	***	−0.36671	***	−0.35424	***	−0.26725	***	−0.24981	***
	LAG3	−0.20658	***	−0.24526	***	−0.305	***	−0.29489	***	−0.32813	***	−0.30943	***
	HAVCR2	−0.19096	***	−0.22682	***	0.060336	0.164236	0.075085	0.107006	−0.10066	*	−0.09005	0.0662
	GZMB	−0.23249	***	−0.26123	***	−0.26768	***	−0.27517	***	−0.07778	0.082918	−0.06375	0.193861

*Note:* *p < 0.05, **p < 0.01, ***p < 0.001.

Abbreviations: BLCA, bladder urothelial carcinoma; Cor, R value of Spearman's correlation; KIRC, kidney renal clear cell carcinoma; None, correlation without adjustment; Purity, correlation adjusted by purity; PRAD, prostate adenocarcinoma; TAM, tumor‐associated macrophage; Tfh, Follicular helper T cell; Th, T helper cell; Treg, regulatory T cell.

### 3.8 Genetic Alterations and Expression of DHCR7 in Urogenital Cancers

**FIGURE 10:**
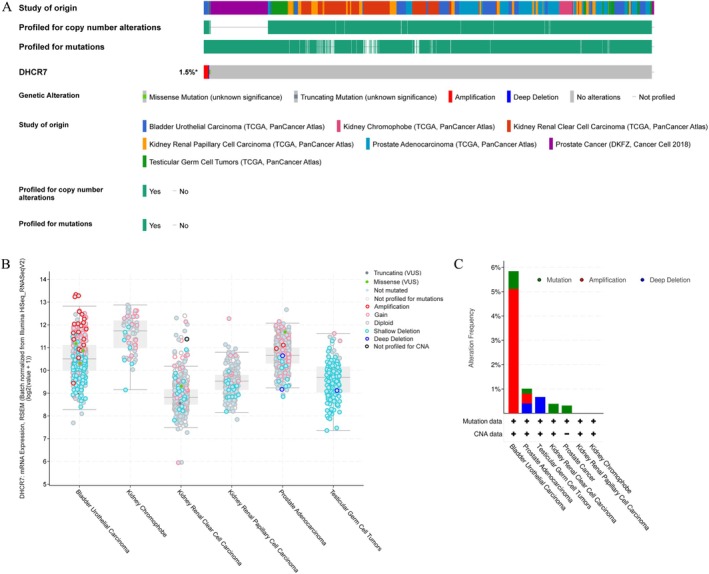
DHCR7 genomic alterations in six urogenital cancers analyzed by the cBioPortal database (A–C) (A) OncoPrint of DHCR7 gene alterations in cancer cohort. (Different colors mean different types of genetic alterations and amplification accounts for the largest proportion). (B) main type of DHCR7 gene alterations in cancer groups. (C) Details of DHCR7 gene alteration types in cancer cohorts.

**FIGURE 11:**
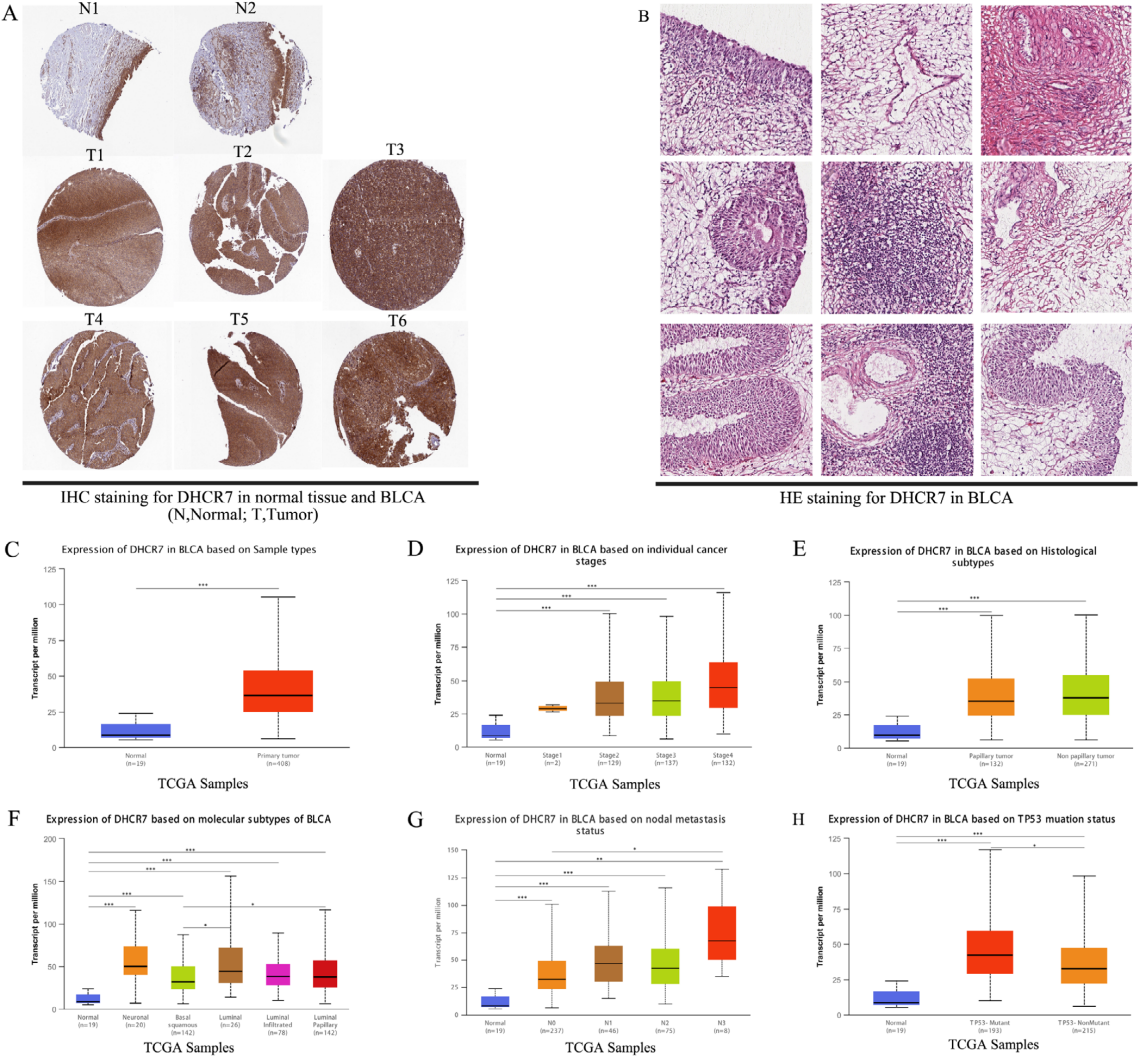
The staining of IHC and HE for DHCR7 in BLCA (A‐B) and DHCR7 differential expression in bladder cancer with different clinical subgroups (C–H) analyzed by the UALCAN database. (A) IHC staining for DHCR7 in normal tissue and BLCA patient tissue analyzed by HPA database; (B) HE staining for DHCR7 in BLCA patient tissue analyzed by HPA database; (C–H) DHCR7 expression between normal(*n* = 19) and BLCA primary tumor(*n* = 408) (C); DHCR7 differential expression in BLCA with individual cancer stages (*n* = 400) (D), histological subtypes (*n* = 403) (E), molecular subtypes (*n* = 408) (F), nodal metastasis status (*n* = 366) (G), TP53 mutation status (*n* = 408) (H) (*p < 0.05, **p < 0.01, ***p < 0.001).

Next, we conducted an analysis of genomic alterations of DHCR7 in urogenital cancers using the cBioPortal website. The results revealed that DHCR7 genomic alterations were present in 1.5% of patients. These alterations encompassed different types, such as missense mutations, truncating mutations, amplifications, and deep deletions (Figure 10A). Interestingly, urogenital cancers exhibited varying levels of gene expression depending on the specific type of DHCR7 gene alteration (Figure 10B). Copy number variations (CNVs) were predominantly observed in BLCA and PRAD, while no CNVs were found in kidney chromophobe and kidney renal papillary cell carcinoma (Figure 10C). To assess DHCR7 expression at the protein level, we obtained immunohistochemical images from the HPA database. Taking BLCA as an example, we observed significantly higher protein expression of DHCR7 in BLCA tissues compared to normal tissues (Figure 11A,B). Additionally, we explored DHCR7 expression in BLCA with different clinical characteristics using the UALCAN database. The data demonstrated significant differential expression of DHCR7 across various factors, including cancer stages, histological subtypes, molecular subtypes, nodal metastasis status, and TP53 mutation status in BLCA (Figure 11C–H). Collectively, these findings indicate that DHCR7 experiences genomic alterations and differential expression in diverse tumor types, underscoring its pivotal role in cancer onset and progression.

### 3.9 Gene Set Enrichment Analysis of DHCR7 in BLCA

**FIGURE 12:**
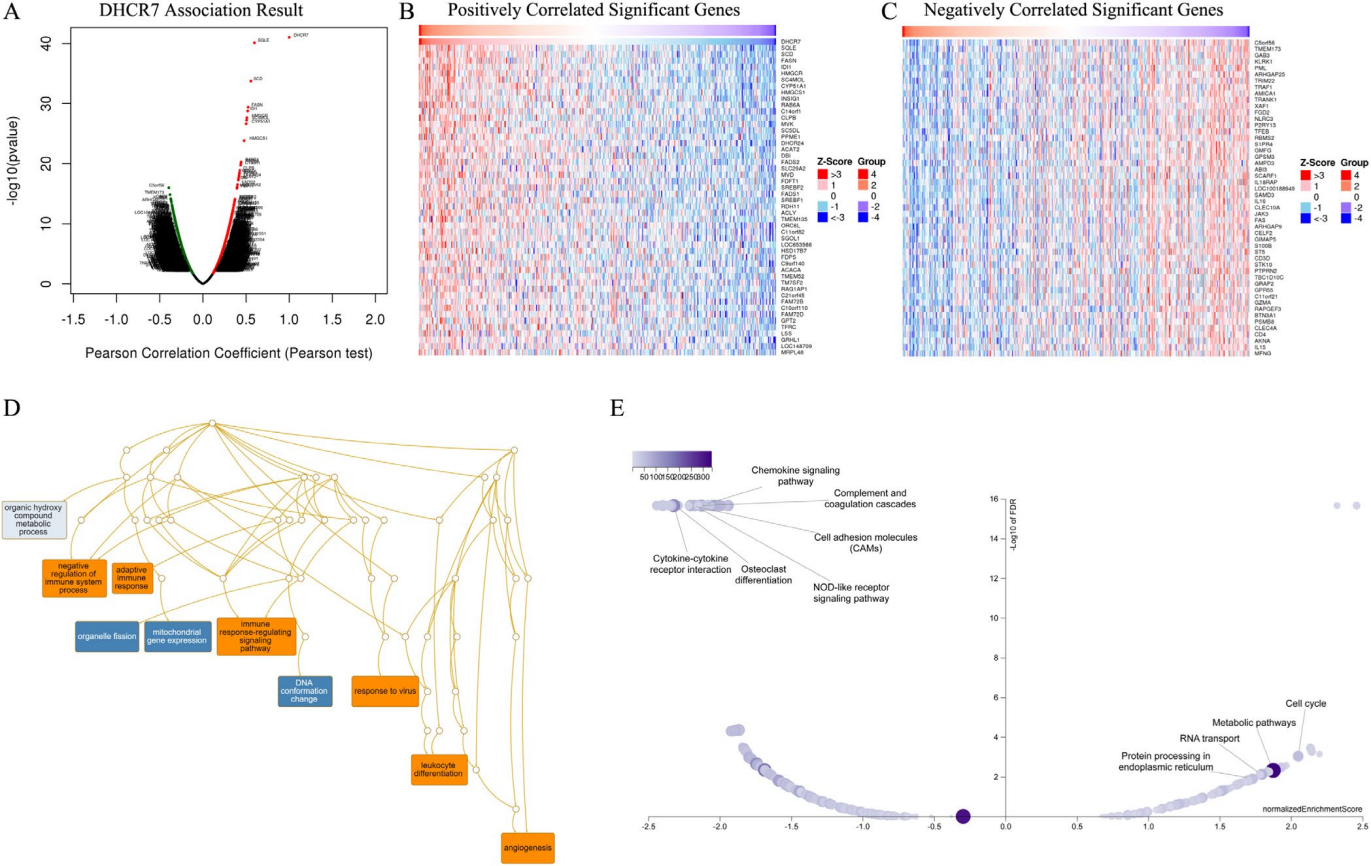
DHCR7 co‐expression genes in BLCA analyzed through the Linked Omics database. (A) Highly correlated genes of DHCR7 tested by the Pearson test in the BLCA cohort. (B, C) Top 50 positive co‐expression genes (B) and negative co‐expression genes (C) of DHCR7 in the heat map in the BLCA; (D) Directed acyclic graph of DHCR7 GO analysis (biological process) in the BLCA cohort. (E) Volcano plot of DHCR7 KEGG pathways in the BLCA cohort.

The above results revealed a significant association between DHCR7 and the prognosis and immunology of cancers. To verify the potential function of DHCR7 in tumor tissue, we explored DHCR7 co‐expression networks using the Linked Omics database. In BLCA, a set of genes (represented by dark red dots) displayed a significant positive correlation with DHCR7, while another set of genes (represented by dark green dots) showed a negative correlation (false discovery rate (FDR) < 0.01) (Figure 12A). A heat map was generated to show the top 50 genes that positively and negatively correlated with DHCR7 (Figure 12B,C). Furthermore, from the Table S1, we can see that SQLE (squalene epoxidase) (*r* = 0.598), SCD (stearoyl‐CoA desaturase) (*r* = 0.556), and FASN (fatty acid synthase) (*r* = 0.524) were three genes which exhibited the strongest association with DHCR7 expression in BLCA (*p* = 7.653e‐41, 1.885e‐34, and 4.095e‐30, respectively). To determine the main gene ontology (GO) terms of DHCR7 co‐expression genes, we employed gene set enrichment analysis (GSEA). Analysis of the biological process categories of GO revealed that DHCR7 and its co‐expression genes primarily participate in the regulation of the immune system process, leukocyte differentiation, virus response, and angiogenesis (Figure 12D). Subsequently, we conducted Kyoto Encyclopedia of Genes and Genomes (KEGG) pathway analysis, which demonstrated that the co‐expressed genes were enriched in cell cycle, metabolic pathways, protein processing in the endoplasmic reticulum, and the chemokine signaling pathway (Figure 12E). These findings suggest that DHCR7 expression may play essential roles in human cancers by regulating the immune response of the TEM.

### 3.10 DHCR7 Is Required for Growth and Proliferation of Renal Cell Carcinoma Cells

**FIGURE 13:**
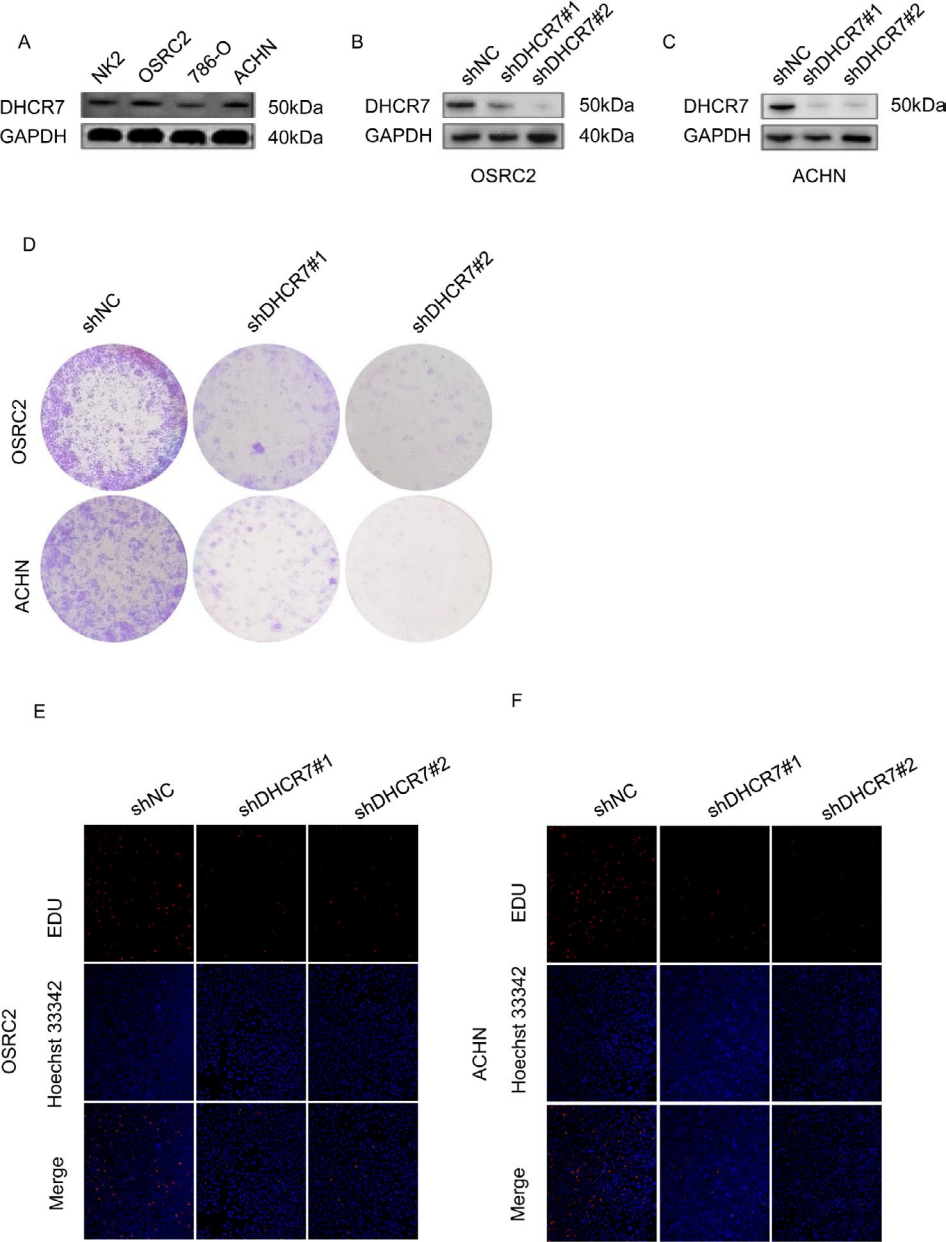
DHCR7 is required for growth and proliferation of renal cell carcinoma cells. (A) Western blotting analysis of DHCR7 levels in NK2, OSRC‐2, 786‐O, and ACHN cells, (B and C) after DHCR7 knockdown in OSRC‐2 and ACHN cell lines. (D–F) Colony formation assay and EdU stain were performed to detect the cell growth and proliferation in OSRC‐2 and ACHN cell lines.

To determine whether DHCR7 is essential for cancer cell growth, we performed genetic knockdown of DHCR7 in a panel of human renal cell carcinoma cell lines using an shRNA system, and examined the effects of DHCR7 knockdown on the proliferation and viability of renal cell carcinoma cells. We observed that DHCR7 was expressed in NK2, OSRC‐2, 786‐O, and ACHN cell lines (Figure 13A), and its expression dramatically downregulated after being transfected by shDHCR7#1 and shDHCR7#2 in OSRC‐2 and ACHN cell lines (Figure 13B,C). We next evaluated the effect of DHCR7 knockdown on growth and proliferation in these cells. As shown in Figure 13D–F, we observed that DHCR7 knockdown significantly suppressed renal cell carcinoma cell proliferation. These results indicate that DHCR7 knockdown inhibits the growth of cancer cells and can be a therapeutic target.

## 4 Discussion

DHCR7 catalyzes the conversion of 7‐dehydrocholesterol (7‐DHC) to cholesterol, thereby regulating cellular sterol homeostasis [23]. Given that cholesterol and its derivatives (e.g., oxysterols) are crucial for membrane integrity, signaling pathways, and post‐translational modifications (e.g., Hedgehog protein maturation), dysregulation of DHCR7 contributes to cancer development through multiple mechanisms. Nowadays, mechanisms of DHCR7 in oncogenesis include: (1) Cholesterol metabolism and cancer cell proliferation. DHCR7 overexpression has been linked to increased cholesterol levels, which may promote tumor growth by: (a) Enhancing lipid raft formation, facilitating oncogenic signaling (e.g., PI3K/AKT, MAPK) [6, 24]. For instance, DHCR7 promotes cervical cancer lymph node metastasis by activating KANK4/PI3K/AKT axis [25]; (b) Supporting steroid hormone synthesis in hormone‐dependent cancers (e.g., breast, prostate) [26, 27]; (c) Providing substrates for prenylation of small GTPases (e.g., Ras, Rho), which drive malignant transformation [28]. (2) Hedgehog (Hh) signaling activation. DHCR7 deficiency leads to 7‐DHC accumulation, which can inhibit Smoothened (SMO), a key component of the Hh pathway. Conversely, DHCR7 upregulation may enhance Hh signaling by reducing 7‐DHC, promoting tumor progression in triple‐negative breast cancer [7, 29]. (3) Oxidative stress and ferroptosis. 7‐DHC is highly susceptible to oxidation, generating reactive oxygen species (ROS) that induce DNA damage. Impaired DHCR7 activity may lead to 7‐DHC accumulation, increasing oxidative stress and genomic instability, thereby driving tumor initiation [30]. Additionally, a recent study found that DHCR7 can promote ferroptosis by metabolizing 7‐dehydrocholesterol (7‐DHC) into cholesterol, thereby reducing cellular 7‐DHC levels. In cancer, DHCR7 inhibition elevates 7‐DHC, suppressing ferroptosis but paradoxically promoting metastasis, while targeting upstream enzymes (e.g., EBP) to deplete 7‐DHC induces ferroptosis and inhibits tumor growth [8, 9]. However, its mechanisms in oncogenesis and tumor immunology remain poorly understood. In this study, we systematically investigated the expression patterns and prognostic significance of DHCR7 across multiple cancer types using diverse datasets. Our findings revealed that DHCR7 is highly expressed in most cancers, and elevated DHCR7 expression is associated with poor prognosis. Notably, our results also demonstrated that DHCR7 influences immune cell infiltration and correlates with immune checkpoint markers, suggesting its pivotal role in tumor immunology. Firstly, we analyzed DHCR7 expression in cancer and normal tissues using the Oncomine, TIMER, GEPIA, and UALCAN databases. The results indicated that DHCR7 is overexpressed in the majority of cancers, with the exception of brain/CNS cancer, cervical cancer, melanoma, and prostate cancer. These findings are consistent with previous studies in bladder cancer and cervical cancer [4, 6]. Next, we examined the relationship between DHCR7 expression and clinical outcomes, including OS, tumor stages, and metastasis. The analysis revealed that high DHCR7 expression is associated with worse OS in BLCA, CESC, HNSC, LIHC, LUAD, PAAD, SARC, UCEC, ACC, UVM, and LUSC. Furthermore, DHCR7 expression was positively correlated with advanced tumor stages and metastasis in pan‐cancer samples, including BLCA, KICH, KIRP, LUAD, LUSC, and TGCT. We also conducted cell experiments to validate DHCR7's effects in cancer cells. DHCR7‐knockdown can inhibit cell growth and proliferation in renal cell carcinoma. These findings underscore the potential of DHCR7 as a prognostic biomarker and its involvement in tumorigenesis. To explore the potential mechanisms, we evaluated DHCR7 expression in immune subtypes and molecular subtypes across various cancers. The results demonstrated that DHCR7 expression levels vary significantly among different immune and molecular subtypes, suggesting its potential role in tumor immunomodulation. Additionally, clinical observations indicate that individual patients exhibit varying responses to immune checkpoint inhibitors (ICIs). Resistance to ICIs can arise from multiple factors, including antigen presentation, TME, tumor‐associated macrophages (TAMs), immunosuppressive metabolites, genetic factors, and biomarker responses [31]. In this study, our data revealed strong correlations between DHCR7 expression and immune checkpoint‐related genes, tumor mutational burden (TMB), microsatellite instability (MSI), neoantigen levels, and mutant allele tumor heterogeneity (MATH) in most human cancers. Moreover, DHCR7 expression was significantly associated with immune cell infiltration in the TME. For instance, DHCR7 exhibited positive effects on the expression of regulatory T cell (Treg) and T cell exhaustion markers, including FOXP3, STAT5B, TGFB1, PDCD1, CTLA4, and LAG3 in BLCA, KIRC, and PRAD. These results suggest that higher DHCR7 expression may contribute to reduced sensitivity to tumor immunotherapy, highlighting the potential for combining DHCR7 inhibitors with therapeutic strategies in future cancer treatments.

## 5 Conclusions

In summary, elevated expression of DHCR7 is significantly associated with advanced tumor stage, metastasis, and unfavorable prognosis across multiple human cancer types. However, the precise molecular mechanisms underlying its involvement in oncogenesis and immunotherapy remain largely unexplored. Further in‐depth mechanistic investigations and preclinical studies are required to elucidate the functional roles of DHCR7 in tumorigenesis and its implications for therapeutic targeting.

## Author Contributions

X.W., W.Z., and L.W. contributed equally to this work. D.L. and Z.W. conceived, initiated, designed, and supervised the study. X.W., W.Z., and L.W. acquired, analyzed, and interpreted the data. X.W. and W.Z. drafted the manuscript, D.L. and Z.W. revised it. All authors gave final approval of the version to be published, and agreed to be accountable for all aspects of the work.

## Ethics Statement

The authors have nothing to report.

## Consent

All authors have provided their consent for publication.

## Conflicts of Interest

The authors declare no conflicts of interest.

## Supporting information


**Data S1:** cnr270376‐sup‐0001‐supinfo.docx.

**Table S1:** cnr270376‐sup‐0002‐TableS1.xlsx.

## Data Availability

The data that support the findings of this study are available on request from the corresponding author. The data are not publicly available due to privacy or ethical restrictions.
