# Supplementary material for: DHCR7 as a Prognostic and Immunological Biomarker in Human Pan‐Cancer: A Comprehensive Evaluation
Source: Cancer Rep (Hoboken). 2025 Nov 6;8(11):e70376. doi: 10.1002/cnr2.70376 (PMC12591708; doi:10.1002/cnr2.70376)
Supplement: Supplementary file 1 — Data S1: cnr270376‐sup‐0001‐supinfo.docx. [file CNR2-8-e70376-s002.docx]

**DHCR7 as a Prognostic and Immunological Biomarker in Human Pan-Cancer: A Comprehensive Evaluation**

*Xianghua Wu^1^, Weiwei Zheng^2^, Li Wang^1^，Dan Lin^1^*, Zhaoxing Wu^3,4*^*

^1^Department of Neurology, The First People's Hospital of Yuhang District, Hangzhou, 311199, China.

^2^Department of Laboratory Medicine, The First Affiliated Hospital of USTC,

Division of Life Science and Medicine, University of Science and Technology of China, Hefei, Anhui, 230001, China.

^3^Department of Hematology (Key Laboratory of Cancer Prevention and Intervention, China National Ministry of Education), The Second Affiliated Hospital, College of Medicine, and ^4^Cancer Institute, Zhejiang University, Hangzhou, 310009, China.

**^*^Corresponding authors:**

E-mail: [13732270615@163.com](mailto:13732270615@163.com) (D.L). [zywzx@zju.edu.cn](mailto:zywzx@zju.edu.cn) (Z.X.W).

**
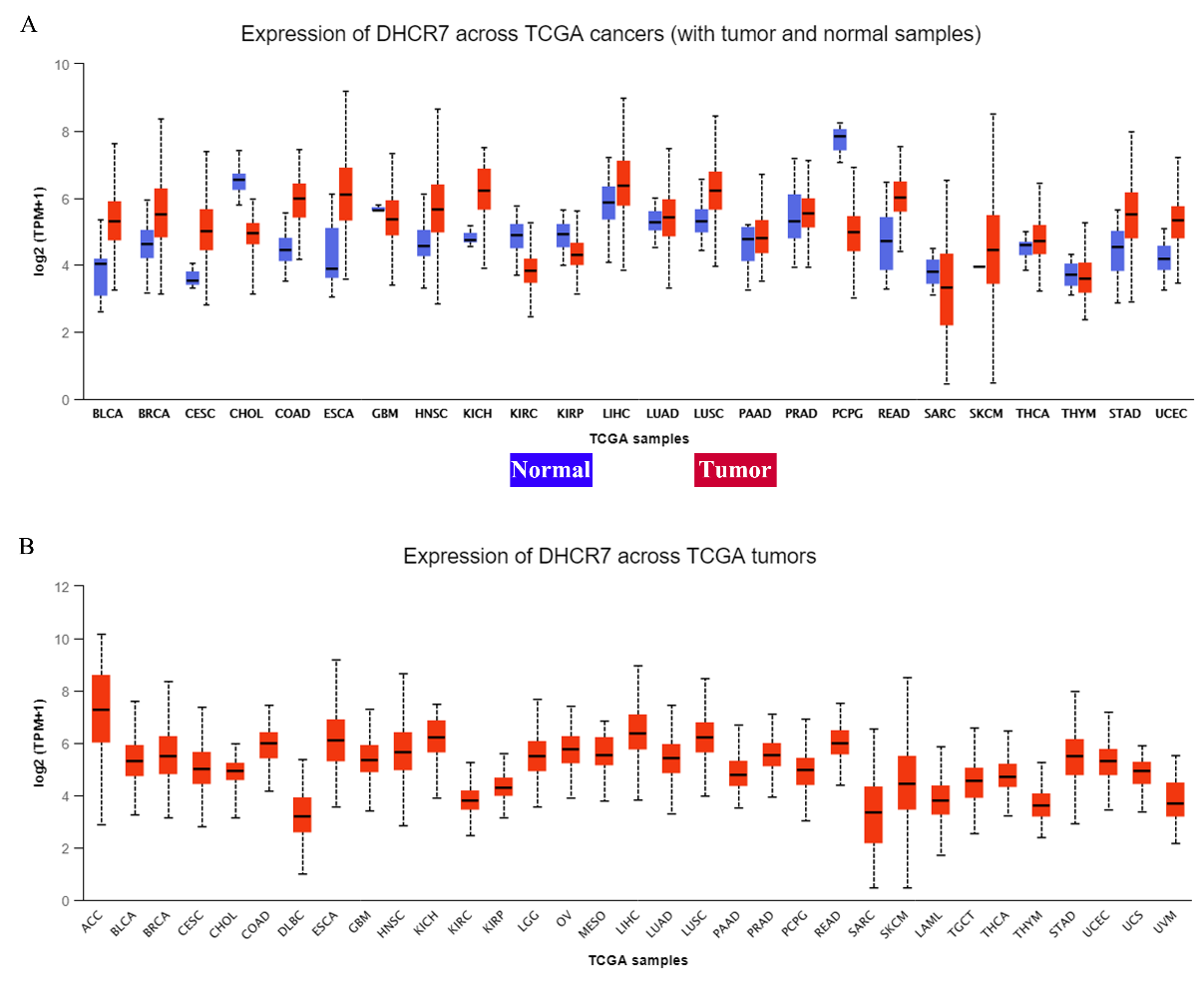
**

**S.Fig.1** **The mRNA expression of DHCR7 in pan cancers analyzed by UALCAN.** (A) DHCR7 expression in different cancers and associated normal tissue from TCGA samples analyzed by UALCAN database. (B) DHCR7 expression levels in different cancer types from the TCGA tumors analyzed by the UALCAN database.


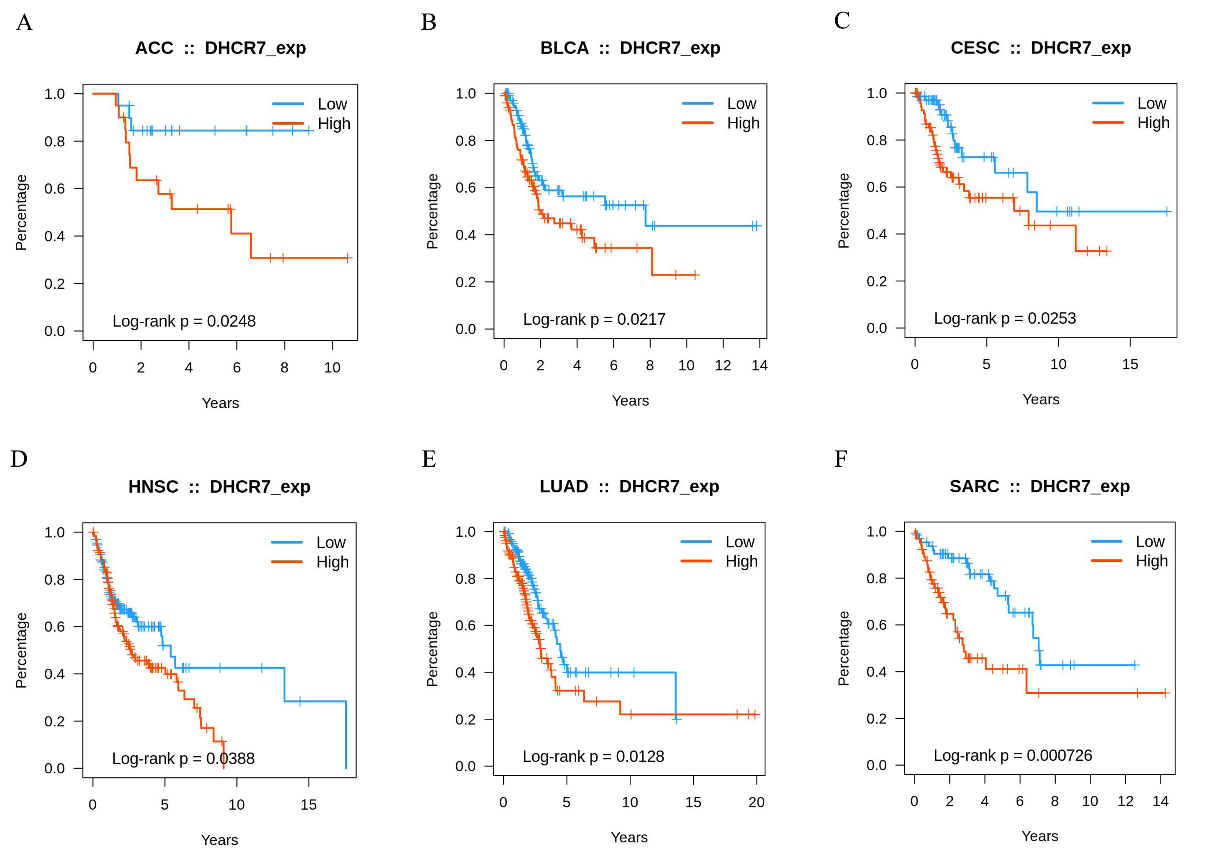


**S.Fig.2 Kaplan-Meier survival curve of OS in human cancers with high and low DHCR7 expression analyzed by the TISIDB database**. High DHCR7 expression was related to worse OS in ACC (A), in BLCA (B), in CESC(C), in HNSC(D), in LUAD(E), and in SARC(F). OS, overall survival.

**
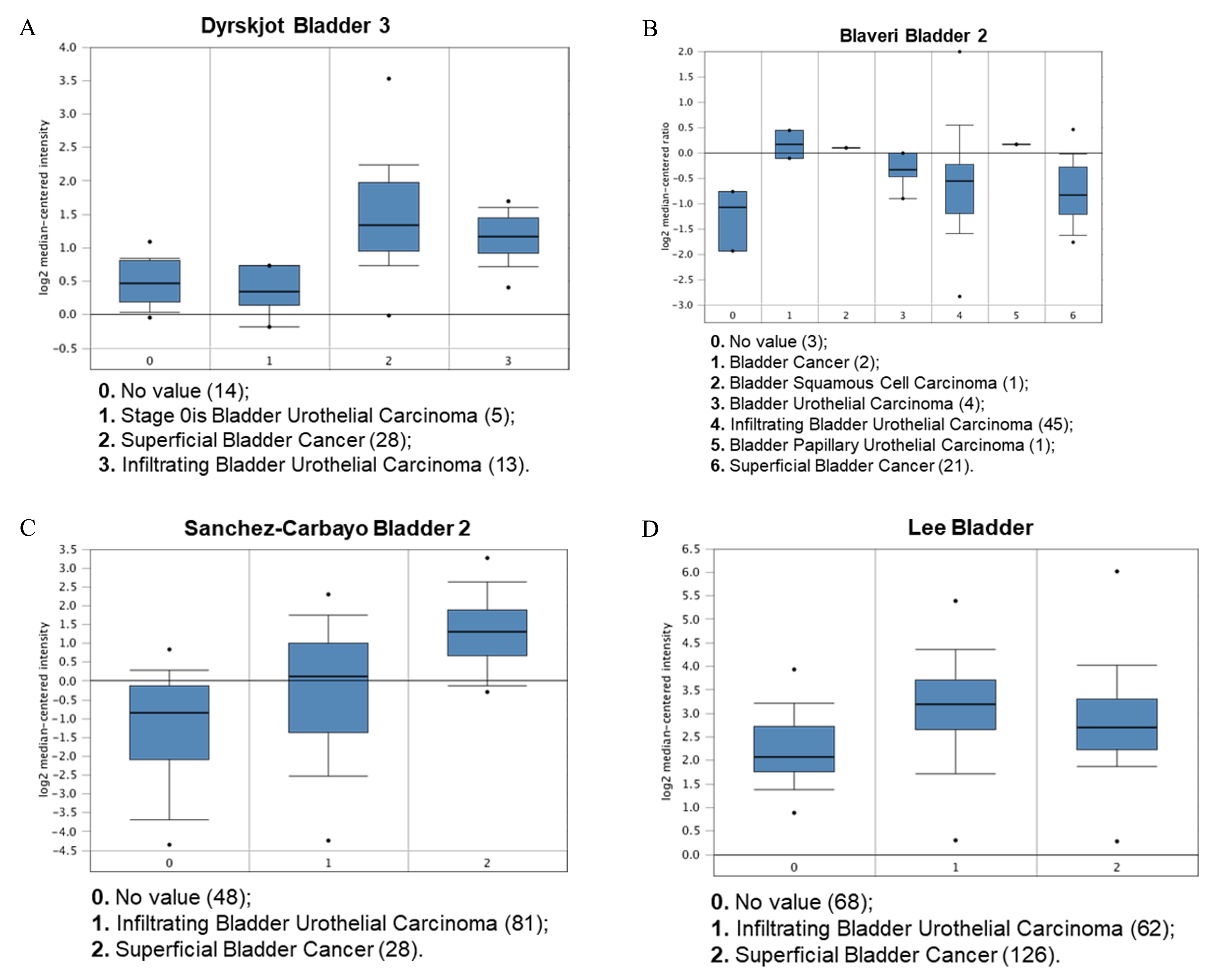
**

**S.Fig.3 The relationship between DHCR7 expression and BLCA stages was identified from Oncomine database.** Box plot shows DHCR7 mRNA level in, respectively, Dyrskjot Bladder 3(A), Blaveri Bladder 2(B), Sanchez-Carbayo Bladder(C), Lee Bladder(D) datasets were significantly higher in BLCA cells than in paired normal cells.
